# Identifying and expression analysis of WD40 transcription factors in walnut

**DOI:** 10.1002/tpg2.20229

**Published:** 2022-07-29

**Authors:** Shuwen Chen, Dapei Li, Sisi Chen, Jianing He, Zengbin Wang, Guiyan Yang, Zhoumin Lu

**Affiliations:** ^1^ Laboratory of Walnut Research Center, College of Forestry Northwest A & F Univ. Yangling Shaanxi 712100 China; ^2^ Key Laboratory of Economic Plant Resources Development and Utilization in Shaanxi Province, College of Forestry Northwest A & F Univ. Yangling Shaanxi 712100 China; ^3^ College of Forestry Northwest A & F Univ. Yangling Shaanxi 712100 China

## Abstract

Walnut (*Juglans regia* L.) is an important woody oil plant and will be affected by abiotic and biological stress during its growth and development. The WD‐repeat (WD40) protein is widely involved in plant growth, development, metabolism, and abiotic stress response. To explore the stress response mechanism of walnut, based on the complete sequencing results of the walnut genome, this study identified and analyzed the physiological, biochemical, genetic structure, and conservative protein motifs of 42 *JrWD40* genes, whose expression to abnormal temperature were tested to predict the potential biological function. The results showed that the open reading frame (ORF) of these*WD40* genes were 807–2,460 bp, encoding peptides were 29,610.55–90,387.98 Da covering 268–819 amino acids, as well as 12–112 phosphorylation sites. JrWD40 proteins were highly conserved with four to five WD40 domains and shared certain similarity to WD40 proteins from *Arabidopsis thaliana* (L.) Heynh. *JrWD40* genes can be induced to varying degrees by low and high temperature treatments. *JrWD40*‐*32*, *JrWD40*‐*27*, *JrWD40*‐*35*, and *JrWD40*‐*21* are affected by high temperature more seriously and their expression levels are higher; while *JrWD40*‐*37*, *JrWD40*‐*26*, *JrWD40*‐*20*, *JrWD40*‐*24*, and other genes are inhibited under low temperature stress. *JrWD40*‐*40*, *JrWD40*‐*28*, and *JrWD40*‐*18* were first suppressed with low expression, while as the treatment time prolonging, the expression level was increased under cold condition. *JrWD40*‐*14*, *JrWD40*‐*18*, *JrWD40*‐*34*, and *JrWD40*‐*3* displayed strong transcriptions response to both heat and cold stress. These results indicated that *JrWD40* genes can participate in walnut adaptation to adversity and can be used as important candidates for walnut resistance molecular breeding.

## Core Ideas

- The woody plant (walnut) WD40 protein was identified.
- Whether the walnut WD40 transcription factor responds to abiotic stress was investigated.
- Some WD40 transcription factors were significantly induced by temperature stress.

**Abbreviations**
ABA: abscisic acid
cDNA: complementary DNA
ORF: open reading frame
pI: isoelectric point
qRT‐PCR: quantitative real‐time PCR
ROS: reactive oxygen species
TF: transcription factor
WD40: WD‐repeat

## 1 INTRODUCTION

Walnut (*Juglans regia* L.), hereafter referred to simply as ‘walnut’, is an important woody, oil‐bearing economic tree and plays an important role in regional economic development (Martínez et al., 2008; Yang et al., 2019). Walnut nuts are rich in fat, protein, amino acids, and mineral elements and have high nutritional and economic value (Abdallah et al., 2015; Yang et al., 2022). In China, walnut trees are planted widely and mainly in Yunnan, Shaanxi, Shanxi, and Xinjiang, covering an area of ∼3.5343 million ha with an annual output of nearly 700,000 t (Feng et al., 2006). However, the mismatch of yield and cultivated area limits the role of walnut in regional economic development; this was mainly due to the impact of climate and other adverse factors in the growth and development process (Vahdati et al., 2009; Wang et al., 2021; Yang et al., 2022). Therefore, in order to improve the efficiency of the walnut industry, it is necessary to reveal the relevant mechanism of walnut adversity response.

The mechanism of stress response involves many aspects including the regulation of growth and development, the adjustment of plant hormones and metabolism, the osmotic response, the setup of membrane protection substances and the balance of reactive oxygen species (ROS), the genes that are mainly involved in conditioning, and the mechanism of stress information transmission in plants (Yang et al., 2021; Zhang et al., 2011). Among them, the transcription factors (TFs) are the most critical type of regulatory factors in the stress regulatory mechanism.

A transcription factor, also known as a *trans*‐acting factor, is a DNA binding protein that can specifically interact with the *cis*‐acting element in the promoter region of the eukaryotic gene (Liu et al., 1999; Yang et al., 2022). It has activation or inhibition effects during the process of transcription. Among the structural components, it mainly consists of four functional domains: a DNA binding domain, a transcriptional regulation domain, an oligomerization site, and a nuclear localization signal (Chen, 1997). It has been found that many TF members belonging to the protein family APETALA2 (AP2)/ethylene‐responsive‐element‐binding protein (EREBP), myeloblastosis (MYB), WRKY (pronounced ‘worky’), NAC (NAM, ATAF1,2, CUC2), basic‐domain leucine‐zipper (bZIP), WD‐repeat (WD40) protein, and other proteins participate in plant abiotic stress response (Hongyan et al., 2016). Among them, WD40 TF family is widely found in eukaryotes and participates in plant development and physiological process and regulates the assembly of multiple complexes in plant resistance and abiotic stress response (Ji et al., 2008; Zhu et al., 2008). The WD40 protein was first isolated and identified in the β subunit of the G protein (a guanine nucleotide regulatory protein) and CDC4 protein (Fong et al., 1986; Xu & Min, 2011). The WD40 protein family is a highly conserved protein family, which generally contains 4 to 16 *cis*‐repeated WD motifs starting from glycine–histidine dimer peptide and ending with tryptophan–aspartate dimeric peptide (Trp‐Asp, WD); each motif contains about 40 amino acid residues (Grigorieva & Shestakov, 1982; Tan et al., 2021). The repeat sequence of the WD40 protein is capable of forming a β helix structure, and this structure is capable of stably or reversibly bind with other proteins or cellular components and provide a rigid platform for interaction (Mishra et al., 2012; Xu & Min, 2011; Smith et al., 1999).

The stable binding platform enables the WD40 protein to combine with basic helix–loop–helix (bHLH) and MYB proteins to form an MYB–bHLH–WD40 complex, which acts as a positive regulator to participate in the synthesis of plant anthocyanin (Xu et al., 2013; Zhang & Schrader, 2017). Studies have shown that WD40 protein with similar functions has been isolated and identified in many plants, and the role of MYB–bHLH–WD40 complex in plants such as MdTTG1 protein in apple [*Malus domestica* (Suckow) Borkh.] (An et al., 2012), PFWD protein in perilla [*Perilla frutescens* (L.) Britton var WD], and MrWD40‐1 protein in bayberry [*Myrica rubra* (L.) Sieb.et Zucc.] have been verified (Yamazaki et al., 2003; Liu et al., 2013). Similarly, the plant WD40 TFs also play vital role in response to abiotic stress. For instance, the *LbTTG1* is derived from *Limonium bicolor* (Bunge) Kuntze and participates in the development of the *Arabidopsis* epidermis and improves the salt tolerance of transgenic *Arabidopsis* by reducing ion accumulation and increasing osmotic pressure (Yuan et al., 2019). Inhibiting *OsRACK_1_A* [The receptor for activated C kinase 1 in rice (*Oryza sativa* L.)] can enhance the tolerance of rice to salt stress and accumulate a large amount of abscisic acid (ABA) (Zhang et al., 2018). Cadmium stress induced the *WDR5a* expression by inhibiting auxin transport and synthesis in *Arabidopsis*, thereby promoting nitric oxide (NO) accumulation and inhibiting root meristem growth (Zhang et al., 2020).

Although the *WD40* family genes play important roles in the regulation of plant stress responses, few studies have been conducted on *WD40* genes in woody plants, especially in walnut, which limit the effective understand to adversity adaptation mechanism of walnut. This is unfavorable to the management of walnut plants and gardens and further affects the benefits of the walnut industry. Therefore, in this study, we identified the WD40 TFs from walnut and analyzed its basic biological information, which will provide important theoretical basis and candidate genes for walnut stress response.

## 2 MATERIALS AND METHODS

### 2.1 Plant materials and treatments

Two‐year‐old ‘Xiangling’ walnut grafted potted seedlings were used as materials. The temperature in the greenhouse was 22±2 °C, and the relative humidity was 70±5%. Seedlings with similar morphology were selected for further experiments. The selected seedlings were subjected to stress treatment at low temperature (6 °C) and high temperature (42 °C), and leaves were collected at 0, 1, 3, 6, and 12 h for each treatment. The seedlings used in the control were placed at room temperature (25 °C) to grow. The collected leaf samples were immediately frozen in liquid nitrogen and stored at −80 °C. All samples were used for total RNA isolation and quantitative real‐time polymerase chain reaction (qRT‐PCR) analysis. Each treatment was set three replicates, and each replicate contained five plants.

### 2.2 Identification, chromosomal location, and bioinformatics analysis of *WD40* genes in walnut

The sequences of *WD40* family genes of walnut were selected from the ‘Xiangling’ walnut transcriptome (not published), and the open reading frame (ORF) was confirmed using ORF finder (http://www.ncbi.nlm.nih.gov/gorf/gorf.html). Candidate *WD40* sequences with completed ORFs were aligned with BLAST (http://blast.ncbi.nlm.nih.gov/Blast) to reconfirm whether they are members of the WD40 family. The obtained WD40 protein sequence was submitted to the online tool ExPASy (http://www.expasy.org/tools/protparam.html) for estimation of amino acid number, molecular weight, and theoretical isoelectric point (pI). Multiple sequence alignment was performed using Clustal X2.0. The chromosomal location information of 42 *JrWD40* genes in the walnut genome *(J. microcarpa* × *J. regia*) was obtained from NCBI (https://www.ncbi.nlm.nih.gov/genome/?term=txid2249226orgn) (Zhu et al., 2019).

**Core Ideas**

- The woody plant (walnut) WD40 protein was identified.
- Whether the walnut WD40 transcription factor responds to abiotic stress was investigated.
- Some WD40 transcription factors were significantly induced by temperature stress.

### 2.3 Phylogenetic analysis of the WD40 family proteins

The MEGA software was used to build a rootless adjacency phylogenetic tree based on the method of neighbor‐joining (Li et al., 2014). Phylogenetic tree setting parameters are as follows: bootstrap method, p‐distance model, partial deletion, uniform rates, and 1,000‐bootstrap test. The other parameters were system default values. Save the constructed phylogenetic tree as Newick format for output and use iTOL (https://itol.embl.de/upload.cgi) online software for beautification editing.

### 2.4 Conserved base sequence analysis of WD40 proteins in walnut

The conservative motif of WD40 protein sequence was analyzed using TBtools (Chen et al., 2020). The minimum length of the conservative motif is six, the maximum length is 50, and the number of the conservative motif is found to be 20. The other parameters are the system default values. Batch‐CD search in NCBI (https://www.ncbi.nlm.nih.gov/Structure/cdd/wrpsb.cgi) was used to search online for the conserved domains. TBtools was used to open the obtained motif, domain, and visualize tree files. To view the overlap of motifs and domain more intuitively, the two results were adjusted and merged together using the TBtools software. Protein secondary structure was predicted using TBtools software and SMART (http://smart.embl.de/smart/set).

### 2.5 Expression analysis of *JrWD40* genes

The total RNA of each sample was extracted by cetyltrimethylammonium ammonium bromide (CTAB) method (Yang et al, 2015). RNase‐free DNase I (Fermentas Inc.) was added into each RNA crude extract to eliminate the remnants DNA under 37 °C for 30 min. The quality and quantity of RNA were assessed by agarose gel electrophoresis and Thermo Scientific NanoDrop One. Then, 0.5 μg RNA was reverse‐transcribed into complementary DNA (cDNA) using PrimeScript RT reagent Kit (CWBIO) under 42°C for 60 min and then 85°C for 5 s. The cDNA was diluted by 10 times and used as the template of qRT‐PCR analysis. The 20.0 μL reaction mixture of qRT‐PCR includes 10.0 μL of SYBR Green Real‐time PCR Master Mix (CWBIO), 2.0 μL cDNA, 1.0 μL forward primers, and 1.0 μL reverse primers. The qRT‐PCR analysis was implemented using CFX96 Touch Real‐Time PCR Detection System (Bio‐Rad Laboratories). The reaction procedure is as follows: 94°C for 30 s, then 45 cycles of 94°C for 12 s, 60°C for 45 s, 72°C for 45 s, and 81°C for 1 s. The *18S rRNA* was used as a reference gene (Xu et al., 2012). Each *JrWD40* gene under different stress treatment conditions was performed in qRT‐PCR assay for three times. All the primers were listed in Supplemental Table S1. The target length of qRT‐PCR products are shown in Supplemental Table S2. The 2^–△△Ct^ method (Livak & Schmittgen, 2001) was used for analysis of the quantitative results, and the relative expression level is relative to the expression of the internal reference gene and the control. The data was processed using SPSS software package. The sample variability is expressed as standard deviation. TBtools (Chen et al., 2020) was used for heatmap drawing, the processed data was imported, the parameters were ‘log scale’, ‘row scale’, and ‘cluster rows’ and ‘zero‐to‐one’ was selected for scale method. Under the same parameter settings, change the ‘tile shape’ option to 'circle’, and then overlap the two panels those showing the same resulting heatmap.

## 3 RESULTS

### 3.1 Sequence characteristics of *WD40* family genes in walnut

**FIGURE 1:**
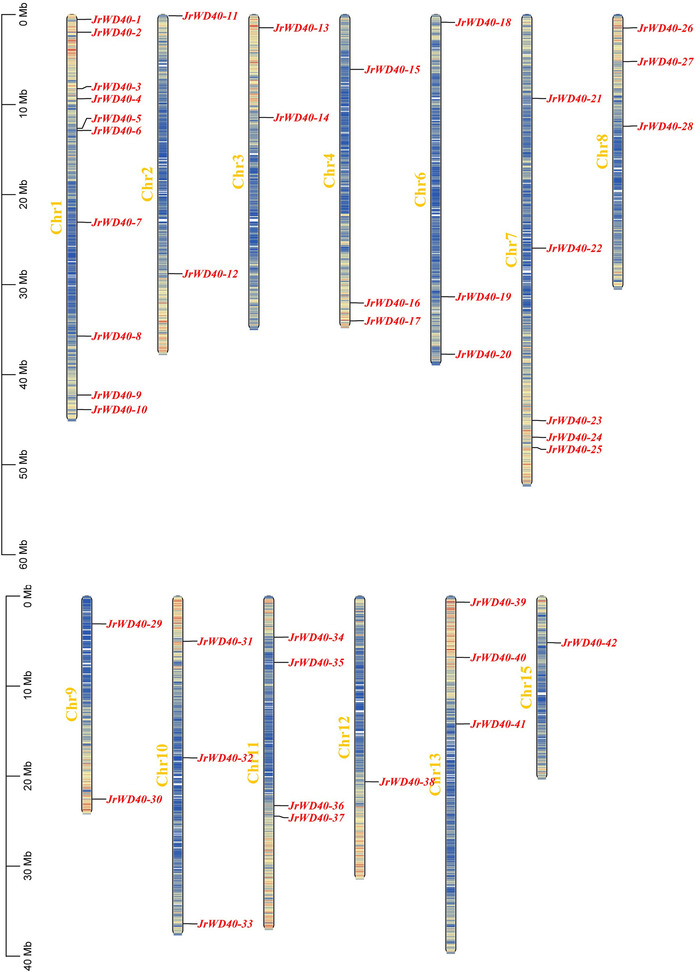
Distribution of the *JrWD40* genes on chromosomes of *Juglans regia*. The chromosome number is shown on the left side of each chromosome

A total of 42 WD40 TFs were screened from the walnut transcriptome. These *JrWD40* genes are distributed on 13 chromosomes of walnut (Chr1, Chr2, Chr3, Chr4, Chr6, Chr7, Chr8, Chr9, Chr10, Chr11, Chr12, Chr13, and Chr15), among which Chr1, Chr7, and Chr11 have the largest number of *JrWD40* genes (10, 5, and 4 genes respectively), while Chr12 and Chr15 contain only one *JrWD40*. The 42 *JrWD40* genes were named according to the order located on the chromosomes (Figure 1). The ORF length of *JrWD40* genes is 807 (*JrWD40‐4*)–2,460 (*JrWD40‐27*) bp, the encoded peptide contains 268–819 amino acids. The molecular weight values range from 29,610.55 to 90,387.98 Da. And the theoretical pI ranges from 4.39 (*JrWD40*‐*18*) to 9.68 (*JrWD40*‐*7*). The average pI of JrWD40 proteins is 6.64, and a small number of WD40 proteins had a low pI. The phosphorylation sites ranged from 12 (*JrWD40*‐*26*) to 112 (*JrWD40*‐*30*) (Table 1).

**TABLE 1:** The sequence characteristics of *Juglans regia* WD40 family genes

Gene name	Accession number	Gene ID from transcriptome	Open reading frame length	No. of amino acids	Molecular weight	Theoretical isoelectric point	Phosphorylation site (Ser/Thr/Tyr)
*JrWD40‐1*	MZ892510	comp9358_c0	1,038	345	38,934.55	4.72	36 (23/7/6)
*JrWD40‐2*	MZ892504	comp30449_c0	1,542	513	57,908.04	6.61	54 (28/19/7)
*JrWD40‐3*	MZ892523	comp30551_c0	1,053	350	38,421.02	4.89	42 (29/12/1)
*JrWD40‐4*	MZ892526	comp25574_c1	807	268	29,610.55	8.02	19 (13/3/3)
*JrWD40‐5*	MZ892513	comp25622_c1	2,001	666	74,465.84	8.06	84 (58/19/7)
*JrWD40‐6*	MZ892539	comp19881_c0	1,359	452	49,393.01	7.67	59 (34/16/9)
*JrWD40‐7*	MZ892528	comp13352_c0	1,359	452	51,970.15	9.68	55 (29/16/10)
*JrWD40‐8*	MZ892507	comp30581_c0	966	321	34,129.55	5.7	33 (23/10/0)
*JrWD40‐9*	MZ892540	comp19284_c0	1,698	565	63,323.12	5.61	51 (35/12/4)
*JrWD40‐10*	MZ892502	comp25439_c1	1,581	526	59,441.32	6.08	51 (33/12/6)
*JrWD40‐11*	MZ892530	comp20808_c0	1,638	545	61,012.25	9.41	64 (49/12/3)
*JrWD40‐12*	MZ892512	comp26723_c1	2,232	743	83,310.85	5.49	82 (63/13/6)
*JrWD40‐13*	MZ892511	comp12443_c0	1,011	336	38,290.14	5.04	38 (21/12/5)
*JrWD40‐14*	MZ892536	comp21767_c0	1,017	338	36,736.67	6.32	40 (29/9/2)
*JrWD40‐15*	MZ892520	comp26647_c0	1,644	547	58,761.54	7.29	82 (57/19/6)
*JrWD40‐16*	MZ892524	comp13738_c0	951	316	35,342.65	5.98	36 (17/17/2)
*JrWD40‐17*	MZ892506	comp26604_c0	1,047	348	38,420.71	4.76	42 (22/17/3)
*JrWD40‐18*	MZ892519	comp31441_c0	1,203	400	42,506.95	4.39	41 (29/11/1)
*JrWD40‐19*	MZ892516	comp10370_c0	1,833	610	66,039.14	6.41	74 (46/25/3)
*JrWD40‐20*	MZ892534	comp26621_c0	1,440	479	53,122.05	8.27	58 (42/11/5)
*JrWD40‐21*	MZ892537	comp28806_c0	1,887	628	69,048.3	5.77	77 (52/16/9)
*JrWD40‐22*	MZ892522	comp21397_c0	1,332	443	50,286.11	5.29	48 (27/14/7)
*JrWD40‐23*	MZ892500	comp48167_c0	1,326	441	47,518.01	8.84	65 (37/21/7)
*JrWD40‐24*	MZ892541	comp21446_c0	1,044	347	38,518.32	8.15	43 (23/18/2)
*JrWD40‐25*	MZ892508	comp27921_c0	1,305	434	46,920.71	8.99	41 (28/13/0)
*JrWD40‐26*	MZ892509	comp26303_c0	2,181	726	78,558.85	6.68	12 (9/3/0)
*JrWD40‐27*	MZ892538	comp28338_c0	2,460	819	90,387.89	6.69	95 (71/18/6)
*JrWD40‐28*	MZ892514	comp28133_c0	1,464	487	54,847.56	7.02	70 (45/15/10)
*JrWD40‐29*	MZ892533	comp17419_c0	1,560	519	58,922.81	9.52	46 (21/16/9)
*JrWD40‐30*	MZ892529	comp28372_c0	1,941	646	70,706.77	5.32	112 (90/14/8)
*JrWD40‐31*	MZ892505	comp10352_c0	1,305	434	47,875.27	5.62	34 (25/7/2)
*JrWD40‐32*	MZ892503	comp26826_c0	1,746	581	64,796.54	5.47	60 (41/15/4)
*JrWD40‐33*	MZ892531	comp14145_c0	1,959	652	71,597.21	5.71	78 (54/17/7)
*JrWD40‐34*	MZ892515	comp14166_c0	969	322	35,301.72	6.7	50 (30/18/2)
*JrWD40‐35*	MZ892518	comp28308_c0	1,488	495	53,823.56	4.49	45 (32/7/6)
*JrWD40‐36*	MZ892527	comp27877_c0	1,554	517	57,928.96	9.55	65 (40/17/8)
*JrWD40‐37*	MZ892525	comp14201_c0	1,167	388	42,715.27	5.23	39 (28/9/2)
*JrWD40‐38*	MZ892535	comp11545_c0	1,014	337	38,260.48	9.25	33 (18/9/6)
*JrWD40‐39*	MZ892517	comp27594_c0	2,199	732	83,376.65	5.89	77 (50/17/10)
*JrWD40‐40*	MZ892521	comp22997_c0	1,485	494	55,496.53	5.82	46 (23/18/5)
*JrWD40‐41*	MZ892501	comp19587_c0	1,257	418	45,443.26	8.41	53 (36/13/4)
*JrWD40‐42*	MZ892532	comp28491_c0	1,347	448	49,575.07	6.73	55 (40/8/7)

### 3.2 Gene sequence similarity and evolutionary relationship of JrWD40 proteins

**FIGURE 2:**
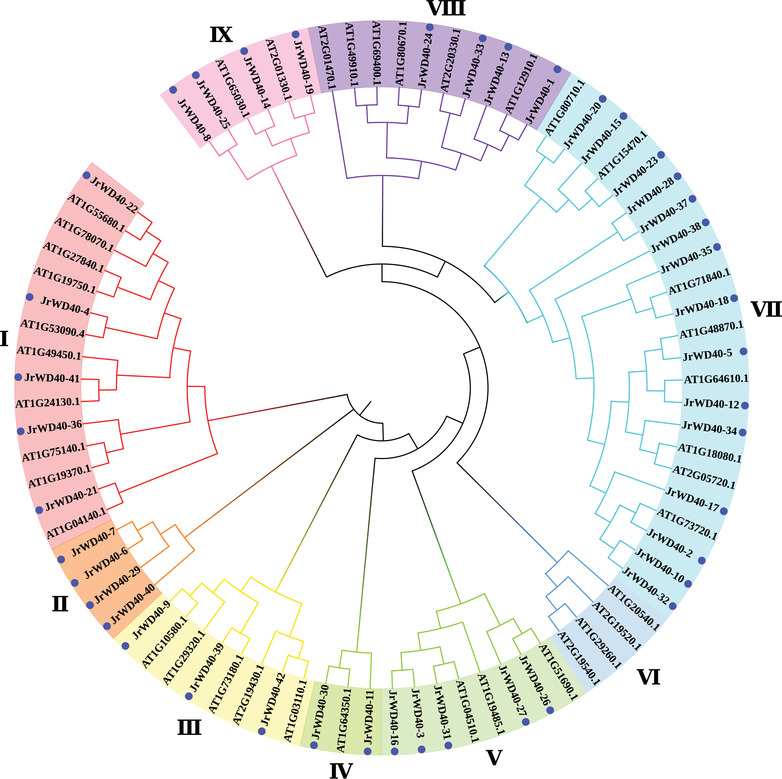
Phylogenetic relationships of WD40 proteins from walnut and *Arabidopsis*. The sequences were aligned at MEGA5 by CLUSTALW, and the rootless phylogenetic tree was derived by neighbor‐joining method. The tree is divided into nine phylogenetic clusters named I–IX. The blue circle was used to mark the location of JrWD40 proteins

Analyzing the protein similarity is helpful to know whether the genes have correlation or similarity in function. Therefore, the sequences of the screened JrWD40 proteins were analyzed and found that the similarity of JrWD40‐25 and JrWD40‐1, JrWD40‐39 and JrWD40‐14 was the highest. JrWD40‐19 shared the lowest similarity with JrWD40‐3 (Supplemental Table S3). To better understand the evolution of walnut WD40 proteins, *Arabidopsis* WD40 family proteins were selected and construct a rootless phylogenetic tree. The results showed that walnut and *Arabidopsis* WD40 proteins could be divided into nine clusters (I, II, III, IV, V, VI, VII, VIII, and IX, including 15, 4, 8, 3, 8, 4, 23, 10, and 6 proteins, respectively) (Figure 2). In each cluster, the walnut and *Arabidopsis* WD40 are orderly distributed in all bunches.

### 3.3 The conserved domain and motif of walnut WD40 proteins

**FIGURE 3:**
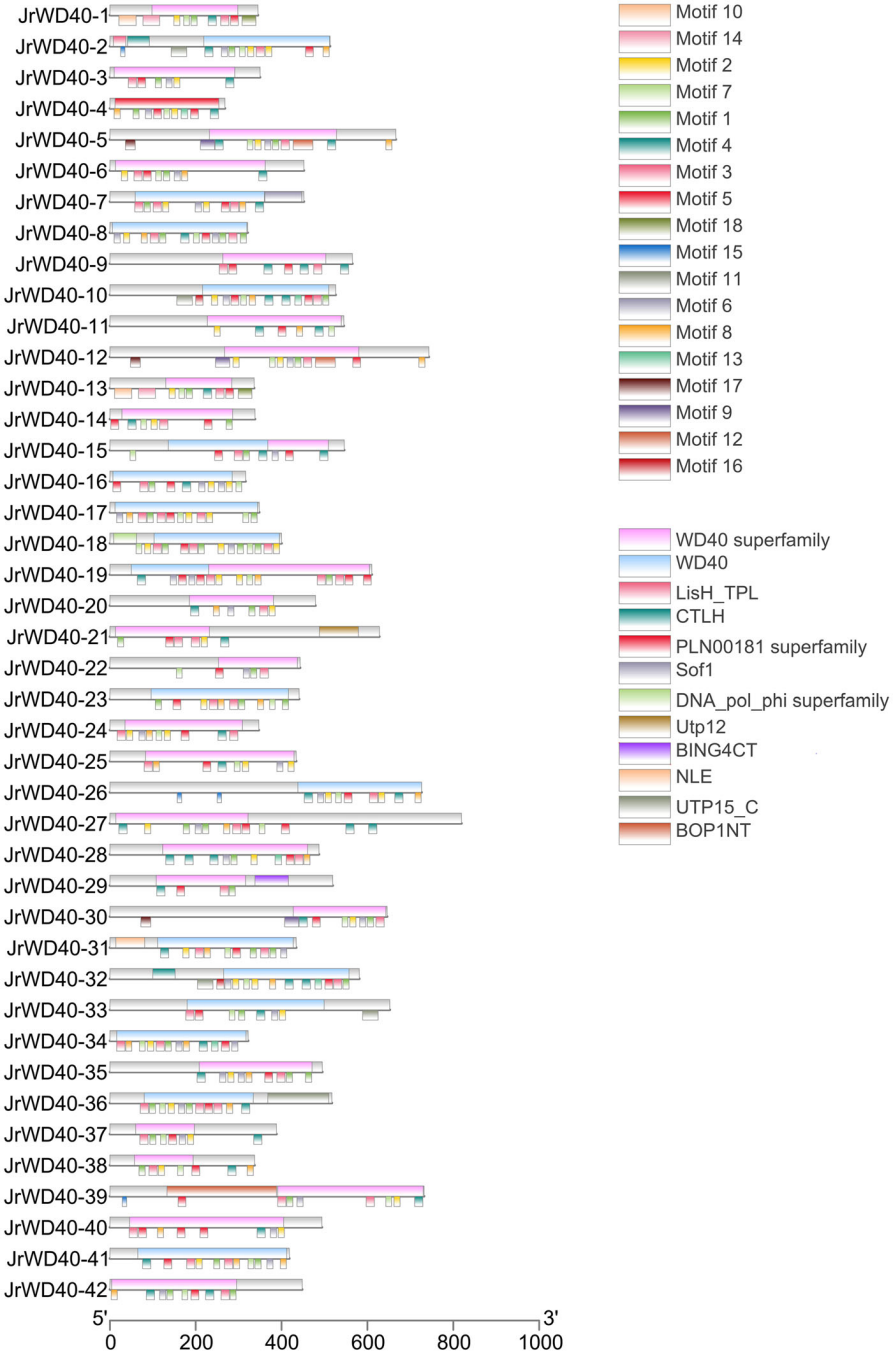
Diversified protein domain and motif structure of JrWD40 proteins. The motif and domain represented by different colors are listed on the right. The domain displayed by each gene is located on the upper half, and the motif is located at the lower half

TBtools was used to analyze the motifs in JrWD40 proteins and their basic information (Figure 3; Supplemental Figure S1, Supplemental Table S4). The results showed that there are 18 different conserved motifs (including 11–50 amino acids) in these 42 JrWD40 proteins, and each JrWD40 protein includes 4 to 15 conserved motifs (Figure 3; Supplemental Table S4). JrWD40‐10, JrWD40‐32, JrWD40‐19, and JrWD40‐18 have the greatest number of conserved motifs, among which JrWD40‐19 contains 15 conserved motifs; JrWD40‐29, JrWD40‐22, JrWD40‐20, and JrWD40‐11 contain four or five conserved motifs. Furthermore, there are five motifs (Motif 1, Motif 5, Motif 4, Motif 2, and Motif 3) are completely conserved in these 42 JrWD40 proteins. Motif 1 is most conserved in all walnut WD40 proteins; Motif 4 and Motif 5 rank second (Figure 3; Supplemental Figure S1).

**FIGURE 4:**
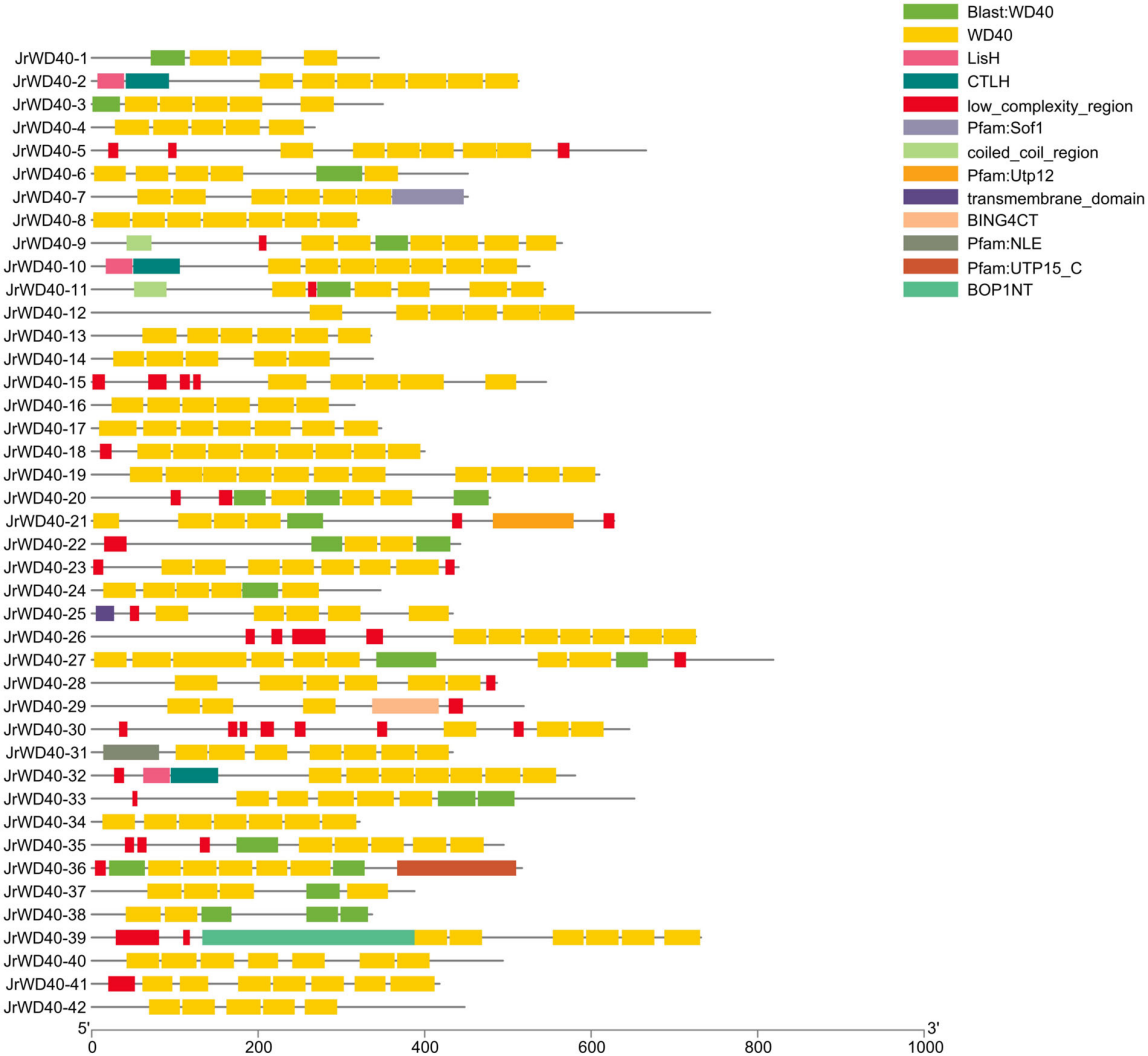
Structure of representative JrWD40 proteins. The protein structure is based on the presence of WD40, and other domains as identified by SMART and TBtools

Except for the WD40 domain, most members of the JrWD40 proteins contain the WD40 superfamily domain, and some contain other known functional domains. The three members (JrWD40‐10, JrWD40‐32, and JrWD40‐2) have both LisH and CTLH domains, while JrWD40‐31 contains NLE domains located in the front of WD40 repeats; JrWD40‐21 and JrWD40‐36 contain Utp12 and UTP15_C in the UTP domain, respectively; JrWD40‐39 contains the BOP1NT domain; the Sof1 domain is located at the end of the WD40 repeat in JrWD40‐7; JrWD40‐29 contains only three WD40 domains and BING4CT domain (Figure 4).

### 3.4 Expression pattern of *JrWD40* genes in response to abnormal temperature stress

Considering that in northwestern China, walnut is more commonly and severely exposed to high temperature and freezing stress, its temperature response mechanism needs to be revealed urgently. At the same time, the *WD40* family gene was reported to be related to temperature response, therefore, in this study, walnut *WD40* family genes were screened, and the expression activity under abnormal temperature conditions was analyzed. The results are as follows.

**FIGURE 5:**
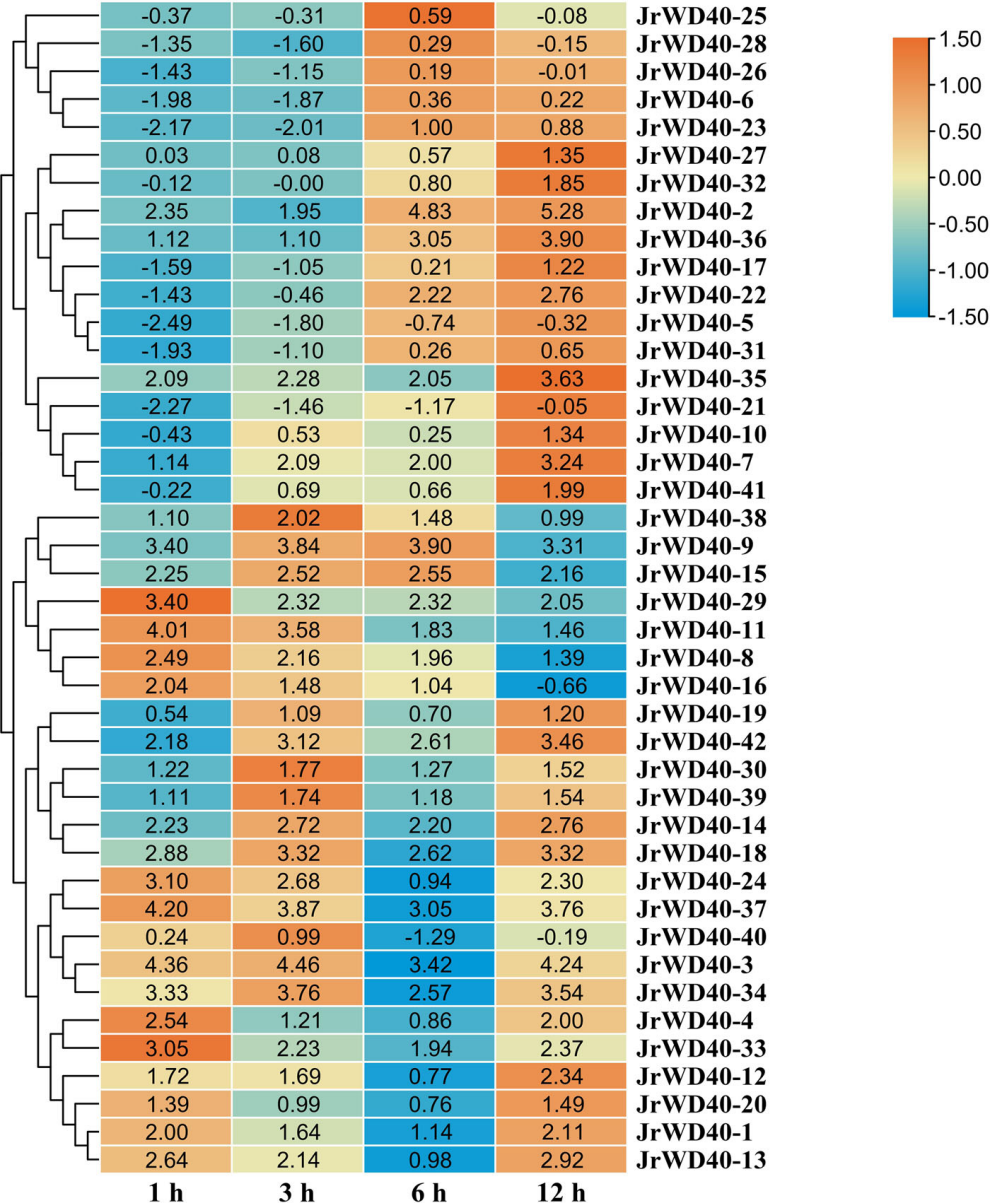
Heatmap representing the expression profiles of the *JrWD40* genes under heat temperature treatment at 0, 1, 3, 6, and 12 h. Numeric values corresponds to the relative expression value. The color scale is displayed on the right. Higher expression levels are shown in orange and lower expression levels are shown in blue

Under high temperature, the expression of *JrWD40‐13, JrWD40‐24*, *JrWD40‐4*, and *JrWD40‐12* showed the same trend, and the expression level decreased gradually with the extension of time and reached the lowest value at 6 h, which was 1.97‐, 1.92‐, 1.82‐, and 1.71‐fold of the control, and then increased at 12 h. The expression of *JrWD40‐40*, *JrWD40‐39*, *JrWD40‐30*, and *JrWD40‐38* changed with time in an ‘N’ shape, which reached the peak at 3 h of stress. The expression trend of *JrWD40‐32* and *JrWD40‐27* was stable and increased with the prolonged stress time, whose highest transcription level was at 12 h (3.61‐ and 2.54‐fold of the control, respectively). The expression of *JrWD40‐10*, *JrWD40‐41*, *JrWD40‐21*, *JrWD40‐35*, *JrWD40*‐*7*, and *JrWD40‐36* increased with the extension of stress time and reached the top level at 12 h. Among them, the expression levels of *JrWD40‐35*, *JrWD40‐7*, and *JrWD40‐36* were stable throughout the stress treatment time, which were 12.35‐, 9.43‐, and 14.93‐fold of control at 12 h, accordingly (Figure 5; Supplemental Figure S2).

**FIGURE 6:**
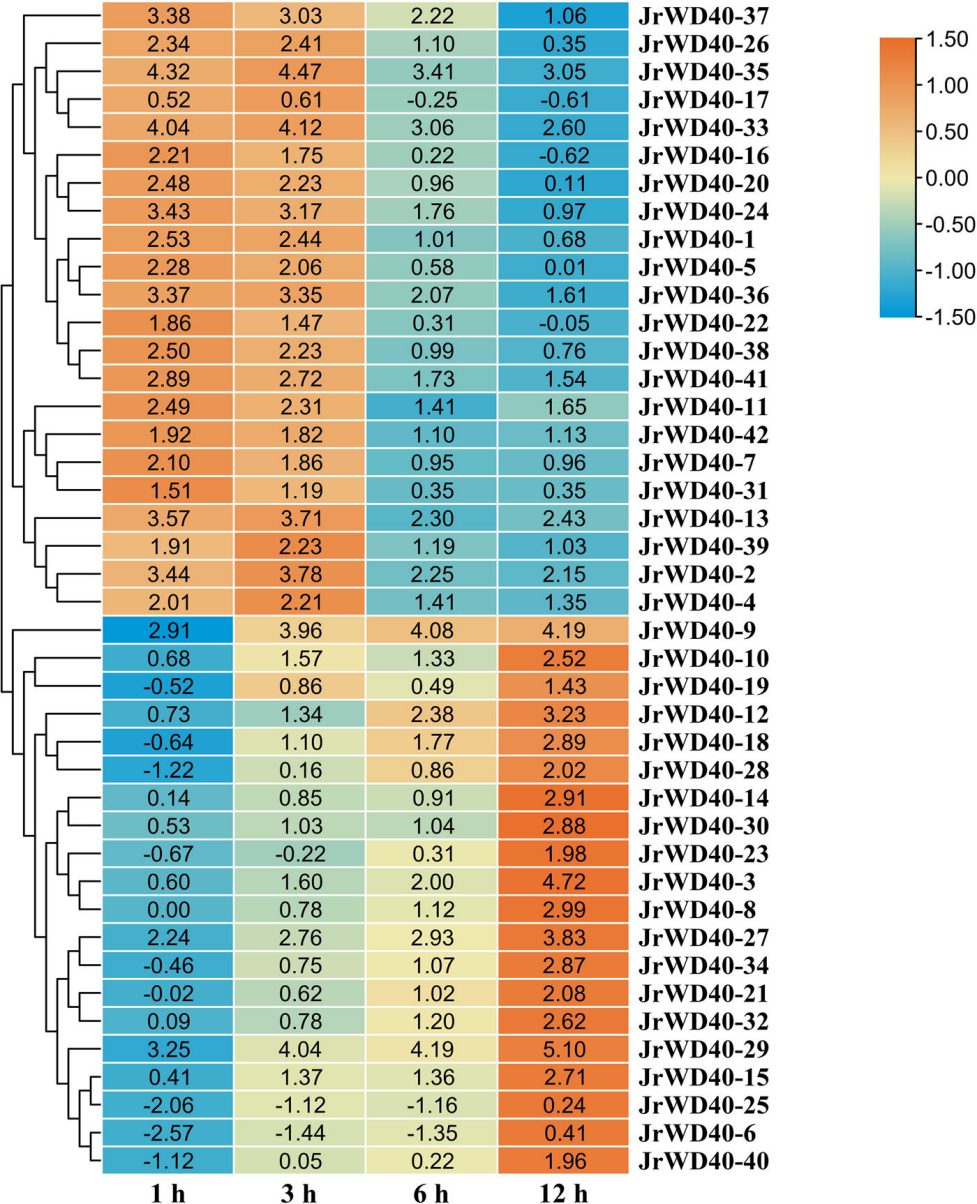
Heatmap representing the expression profiles of the *JrWD40* genes under low temperature treatment

Under low temperature stress, gene expression trend changes can be roughly divided into two categories. The expression levels of 18 genes, including *JrWD40‐30*, *JrWD40‐14*, *JrWD40‐23*, *JrWD40‐3*, *JrWD40‐27*, *JrWD40‐6*, *JrWD40‐19*, *JrWD40‐28*, *JrWD40‐18*, *JrWD40‐8*, *JrWD40‐34*, *JrWD40‐32*, *JrWD40‐21*, *JrWD40‐29*, *JrWD40‐25*, *JrWD40‐15*, *JrWD40‐40*, and *JrWD40‐12* showed an upward trend with time, and their expression was highest at 12 h. The expression levels of *JrWD40*‐*3*, *JrWD40*‐*30*, and *JrWD40*‐*29* at 12 h were 26.42‐, 7.38‐, 34.30‐fold of control, respectively. However, the expression trend of some genes was the opposite. For example, the expression levels of *JrWD40*‐*37*, *JrWD40*‐*26*, *JrWD40*‐*20*, and *JrWD40*‐*16* gradually decreased to 2.08‐, 1.27‐, 1.08‐, and 0.65‐fold of control at 12 h with the extension of stress time. The expression trend of *JrWD40*‐*2*, *JrWD40*‐*13*, *JrWD40*‐*35*, and *JrWD40*‐*4* showed an inverted ‘V’ pattern, and the highest value was appeared at 3 h, which was 13.77‐, 13.12‐, 22.11‐, and 4.63‐fold of control, accordingly. Then the expression level gradually decreased. The expression of *JrWD40*‐*40*, *JrWD40*‐*28*, and *JrWD40*‐*18* was initially suppressed, but the expression level gradually increased with the extension of stress time and reached the maximum level at 12 h. The expression levels of *JrWD40*‐*25* and *JrWD40*‐*6* were inhibited by low temperature stress between 1 and 6 h, and their expression levels returned to normal at 12 h (Figure 6; Supplemental Figure S2).

**FIGURE 7:**
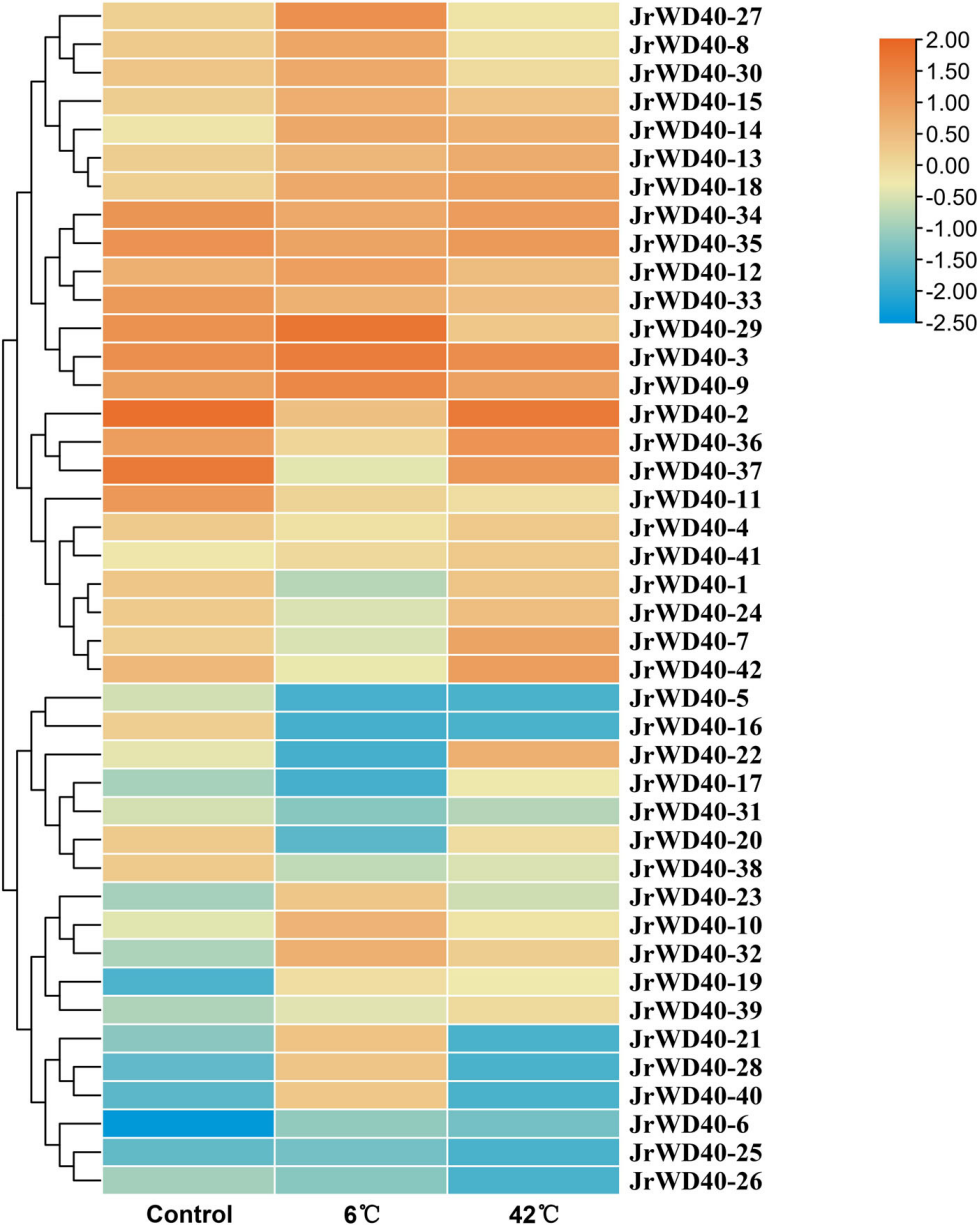
Heatmap of *JrWD40* gene expression profiles after 12 h of heat and low temperature treatment. The nonstress treatment (25 °C) was used as control. The color scale is displayed on the right. Higher expression levels are shown in orange and lower expression levels are shown in blue

Compared with the expression of these *JrWD40* genes under cold and heat conditions, we found that the expression levels of *JrWD40‐29*, *JrWD40‐30*, *JrWD40‐32*, *JrWD40‐27*, and *JrWD40‐9* were higher under low temperature stress, and the expression of these genes were inhibited under high temperature stress during the same treatment time. In contrast, *JrWD40‐2*, *JrWD40‐37*, *JrWD40‐36*, *JrWD40‐7*, and *JrWD40‐42* was normally expressed under high temperature stress, while they were inhibited by low temperature. *JrWD40‐5*, *JrWD40‐16*, *JrWD40‐31*, *JrWD40‐17*, and *JrWD40‐20* were inhibited to varying degrees under two kinds of temperature stresses, and the expression level was relatively low. Interestingly, the transcription of *JrWD40‐32*, *JrWD40‐21*, *JrWD40‐28*, and *JrWD40‐40* was promoted under low temperature stress but was severely inhibited under high temperature stress (Figure 7; Supplemental Figure S2). These results suggested the potential differences among these walnut *WD40* genes in response to abnormal temperature stresses.

## 4 DISCUSSION

Walnut, as an important oil and timber species, not only has high nutritional value, but also has the characteristics of drought resistance and wind resistance (Li et al., 2013). The high quality and yield of walnut is related to variety, location, climate, and other factors. In the northwest of China, severe weather conditions such as long‐term drought, high temperature, and severe cold springs directly affected the yield and quality of walnut (Wu et al., 2016). The research on the nutritional quality of walnut kernels has expanded. Although the research on variety selection, environmental response, and pest control is involved, it is far from enough (Yang et al., 2022; Regueiro et al., 2014; Pollegioni et al., 2012). It is also precisely because of the lack of understanding of walnut adaptation mechanisms that when walnut trees encounter adverse environmental stimuli, such as continuous drought, high temperature, or cold springs, their yields are severely affected, and even large areas of newly planted trees die or whole branches die. This severely restricted the development of the walnut industry. Therefore, the research on the adaptation mechanism of walnut will help to provide strong support for the selection and cultivation of walnut varieties and promote the development of walnut industry.

We have paid attention to TFs that have important regulatory roles, among which, the WD40 family has an important function or role in stress response. Therefore, we focused on this gene family and identified 42 *WD40* genes with complete ORFs from the walnut transcriptome. These WD40 TFs were found to contain WD40 domain or WD40 superfamily domain. So far, most of the WD40 superfamily genes have been identified from *Arabidopsis*, cucumber (*Cucumis sativus* L.), peach [*Prunus persica* (L.) Batsch], millet (*Panicum miliaceum* L.), and rice (Mishra et al., 2014; Ouyang et al., 2012; Steven & Ludwig, 2003; Li et al., 2014; Feng et al., 2019). However, most of the current studies on WD40 family focused on herbs and Gramineae, there are relatively few studies on woody plants. Thus, our results enrich the study of the WD40 family in woody plants. The larger the number of WD40 protein structures, the more conservative and stable. In this study, most JrWD40 proteins contain 4–5 consecutive WD40 protein structures, implying the potential stable and conservative nature of JrWD40 proteins. This is consistent with the conclusion obtained in *Arabidopsis* WD40 proteins, which have similar highly conserved domains as in most eukaryotes (van Nocker & Ludwig, 2003). Considering the views that homology of WD40 proteins from rice and peach shared similar functions to some extent (Feng et al., 2019; Ouyang et al., 2012), the proteins with similar domains and functions were clustered in the same phylogenetic branch (Ouyang et al., 2012; Mishra et al., 2014), the similar motifs shared by WD40 from different species (Supplemental Figure S3) may share common roles (Zhang et al., 2020; Kong et al., 2015; Huang et al., 2008), it is possible that closely related WD40 in the same species may have similar functions. This view is demonstrated in the *Arabidopsis* R2R3MYB subfamily, where closely related proteins can recognize similar target genes and have redundant, overlapping, or cooperative functions (Matus et al., 2008; Dubos et al., 2010). Therefore, based on the topological structure, 42 WD40 TFs are numbered in a counterclockwise order. Considering the structure and conserved domains of the WD40 protein in walnut that we have identified, combined with similar reports from others, we initially believe that some members of these *JrWD40* genes may be related to adversity.

The expression in response to stress can usually effectively predict the stress response function of the target gene. For example, by analyzing the transcription level of walnut WRKY family genes in response to NaCl, PEG_6000_, and abnormal temperature stresses, it is found that *WRKY* family genes have different stress response capabilities, which was further confirmed by transient overexpression method (Yang et al., 2017a; Yang et al., 2017b). *CsWD40* could be induced significantly by ABA and sucrose stress, overexpression of *CsWD40* can supplement the pigments lost in plants induced by sucrose, suggesting that *CsWD40* enhanced the tolerance of green tea [*Camellia sinensis* (L.) Kuntze] plants to osmotic stress by promoting the accumulation of flavonoids (Liu et al., 2018). In wheat (*Triticum aestivum* L.), the plants overexpression of *TaWD40* gene showed much higher germination rate than wild type under the stress of NaCl, mannitol, and ABA (Kong et al., 2015). Under salt stress, the WD40 protein XIW1 (interacting with XPO1) can reduce the accumulation of ABI5 and the sensitivity of ABA response genes to ABA and also reduce the drought stress resistance of Arabidopsis, which is believed to be related to the mechanism of inhibiting seed germination under salt stress (Xu et al., 2019; Cai et al., 2020). Thus, it can be seen that the potential of *WD40* genes to respond to adversity can be predicted by quantitative expression.

In order to confirm whether *JrWD40* genes has the potential to respond to temperature stress, we treated ‘Xiangling’ walnut with low and high temperature stresses to analyze the expression of WD40 TFs. The results showed that nine *JrWD40* genes can be induced by high temperature. Among them, *JrWD40*‐*31, JrWD40*‐*22*, *JrWD40*‐*32, JrWD40*‐*23*, and five other genes have outstanding performance under high temperature stress. The expression of 10 *JrWD40* genes was outstanding under low temperature stress, and the expression level increased gradually with the stress time. It is noteworthy that the expression level of *JrWD40‐32* and *JrWD40‐23* also changed from initial inhibition to obvious expression level under low temperature stress. Finally, the gene expressions of *JrWD40* genes under the two kinds of temperature stress at 12 h were clustered with the control group (25 °C). The results showed that 10 *JrWD40* genes could be induced under low temperature stress, but their expression was severely inhibited under high temperature stress at the same time. However, *JrWD40‐2*, *JrWD40‐22*, *JrWD40‐42*, and *JrWD40‐36* were inhibited at low temperature at the same time, and their expression levels induced by high temperature were nearly consistent with those of the control group. Five genes—*JrWD40‐14*, *JrWD40‐13*, *JrWD40‐15*, *JrWD40‐3*, and *JrWD40‐18*—can be induced under both temperature stresses, and their expression levels are similar to those under normal temperature. Our results are consistent with other results related to the predictive function of gene expression, for instance, *Arabidopsis* WD40 family gene *TTG1* involved in the flavonoid synthesis pathway can be upregulated by low temperature stress (Petridis et al., 2016). Under low temperature or drought stress, *ZFP245* could upregulate the expression of *OSP5C* genes in rice seedlings, and the overexpression lines have higher superoxide dismutase and peroxidase activities than wild type plants, indicating that *ZFP245* may enhance the tolerance of rice to cold and drought stress by regulating the level of proline and removing ROS (Huang et al., 2009). Under the condition of low temperature stress, *ONACo95* inhibition expression lines increased the content of malondialdehyde, accumulated a large amount of ROS, and could regulate the expression of low temperature response genes, which was considered to act as a positive regulator of rice low temperature response (Huang et al., 2016). Considering the results of this study and other reports, we believed that walnut *WD40* genes does have a certain relationship with the temperature response mechanism. Since the response of *WD40* gene to adversity in other studies is presumed to have related functions, and the similar performance of WD40 TFs in this study, we preliminarily believe that these *JrWD40* genes have the function of regulating and participating in walnut response to adversity.

## 5 CONCLUSION

In this study, 42 *JrWD40* genes were identified, and their distribution in gene structure, conserved motifs, and evolutionary relationship were analyzed. *JrWD40* genes are highly stable and conserved. Genes *JrWD40‐14*, *JrWD40‐18*, *JrWD40‐34*, and *JrWD40‐3* were significantly induced by low and high temperature stresses with various expression levels in different stress durations, which can be further explored. The expression level of *JrWD40* genes in ‘Xiangling’ indicates that *JrWD40* genes may play an important role in the resistance of plants to heat and cold stress. This study provides basic information for further research on the function and response mechanism of the *JrWD40* genes.

## AUTHOR CONTRIBUTIONS

Shuwen Chen: Visualization; Writing–original draft and review. Dapei Li: Software. Sisi Chen: Software. Jianing He: Visualization. Zengbin Wang: Software. Zhoumin Lu: Project administration; Visualization; Writing—review & editing. Guiyan Yang: Data curation; Project administration; Resources; Writing—review & editing.

## CONFLICT OF INTERESTS

All the authors declare that they have no conflict of interest.

## Supporting information

Supplemental Table S1. The primers for qRT‐PCR analysis.

Supplemental Table S2. The segment and lengths of qRT‐PCR products of *JrWD40s*

Supplemental Table S3. Sequence similarity characteristics of JrWD40*s*.

Supplemental Table S4. The number and sequence of motifs.

Supplemental Figure S1. Logo for the conserved motifs shared by WD40 proteins in *J. regia*.

Supplemental Figure S2. Histogram showing the expression of walnut *WD40* family genes under high temperature and low temperature stress.

Figure S3. Similar motif analysis of JrWD40s and WD40 from Arabidopsis, wheat, and rice.

## Data Availability

All the data were presented in the main manuscript and additional supporting files.
