# Supplementary material for: Identifying and expression analysis of WD40 transcription factors in walnut
Source: Plant Genome. 2022 Jul 29;15(3):e20229. doi: 10.1002/tpg2.20229 (PMC12806941; doi:10.1002/tpg2.20229)
Supplement: Supplementary file 1 [file TPG2-15-e20229-s001.pdf]

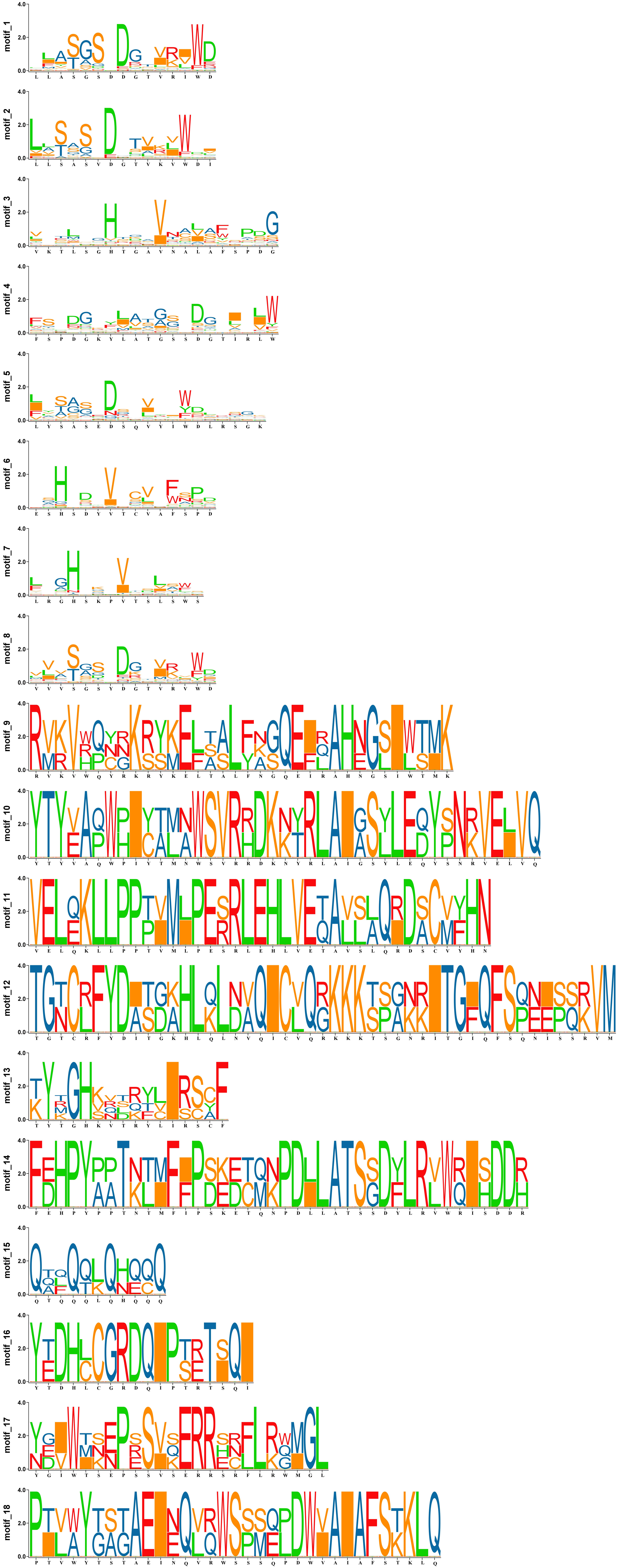
motif_1
L L A S G S D D G T V R I W D
motif_2
L L S A S V D G T V K V W D I
motif_3
V K T L S G H T G A V N A L A F S P D G
motif_4
F S P D G K Y L A T G S S D G T I R L W
motif_5
L Y S A S E D S Q V Y I W D L R S G K
motif_6
E S H S D Y V T C V A F S P D
motif_7
L R G H S K P V T S L S W S
motif_8
V V V S G S Y D G T V R V W D
motif_9
R V K V W Q Y R K R Y K E L T A L F N G Q E I R A H N G S I W T M K
motif_10
Y T Y V A Q W P I Y T M N W S V R R D K N Y R L A I G S Y L E Q Y S N R V E L V Q
motif_11
V E L Q K L L P P T V M L P E S R L E H L V E T A V S L Q R D S C V Y H N
motif_12
T G T C R F Y D I T G K H L Q L N V Q I C V Q R K K K T S G N R I T G I Q F S Q N I S S R V M
motif_13
T Y T G H K V T R Y L I R S C F
motif_14
F E H P Y P P T N T M F I P S K E T Q N P D L L A T S S D Y L R V W R I S D D R
motif_15
Q T Q Q Q L Q H Q Q Q
motif_16
Y T D H L C G R D Q I P T R T S Q I
motif_17
Y G I W T S E P S S V S E R R S R F L R W M G L
motif_18
P T V W Y T S T A E I N Q V R W S S S Q P D W V A I A F S T K L Q
