# Supplementary material for: Identifying and expression analysis of WD40 transcription factors in walnut
Source: Plant Genome. 2022 Jul 29;15(3):e20229. doi: 10.1002/tpg2.20229 (PMC12806941; doi:10.1002/tpg2.20229)
Supplement: Supplementary file 2 [file TPG2-15-e20229-s002.pdf]

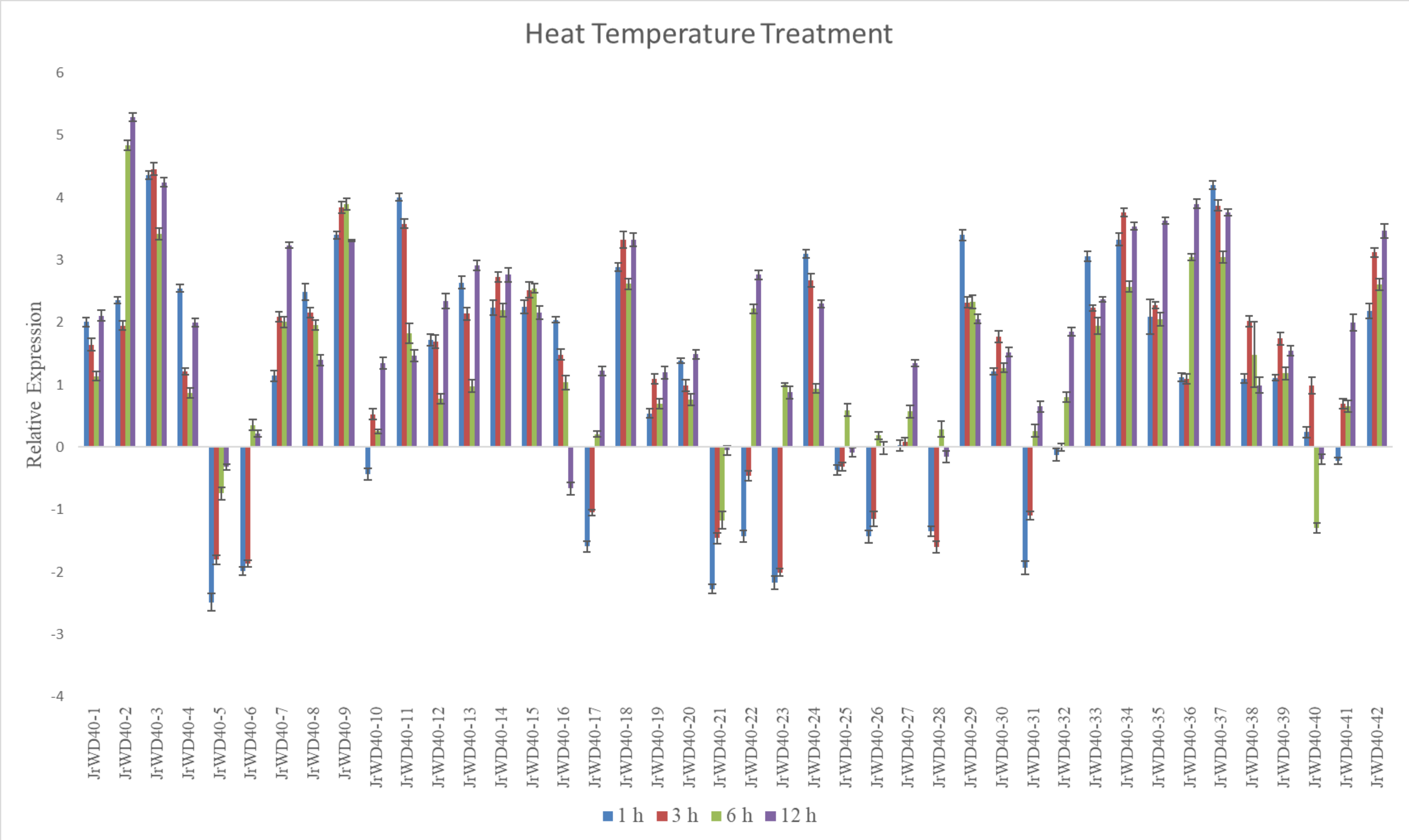
Heat Temperature Treatment
Relative Expression
6
5
4
3
2
1
0
-1
-2
-3
-4
JrWD40-1
JrWD40-2
JrWD40-3
JrWD40-4
JrWD40-5
JrWD40-6
JrWD40-7
JrWD40-8
JrWD40-9
JrWD40-10
JrWD40-11
JrWD40-12
JrWD40-13
JrWD40-14
JrWD40-15
JrWD40-16
JrWD40-17
JrWD40-18
JrWD40-19
JrWD40-20
JrWD40-21
JrWD40-22
JrWD40-23
JrWD40-24
JrWD40-25
JrWD40-26
JrWD40-27
JrWD40-28
JrWD40-29
JrWD40-30
JrWD40-31
JrWD40-32
JrWD40-33
JrWD40-34
JrWD40-35
JrWD40-36
JrWD40-37
JrWD40-38
JrWD40-39
JrWD40-40
JrWD40-41
JrWD40-42
1 h
3 h
6 h
12 h

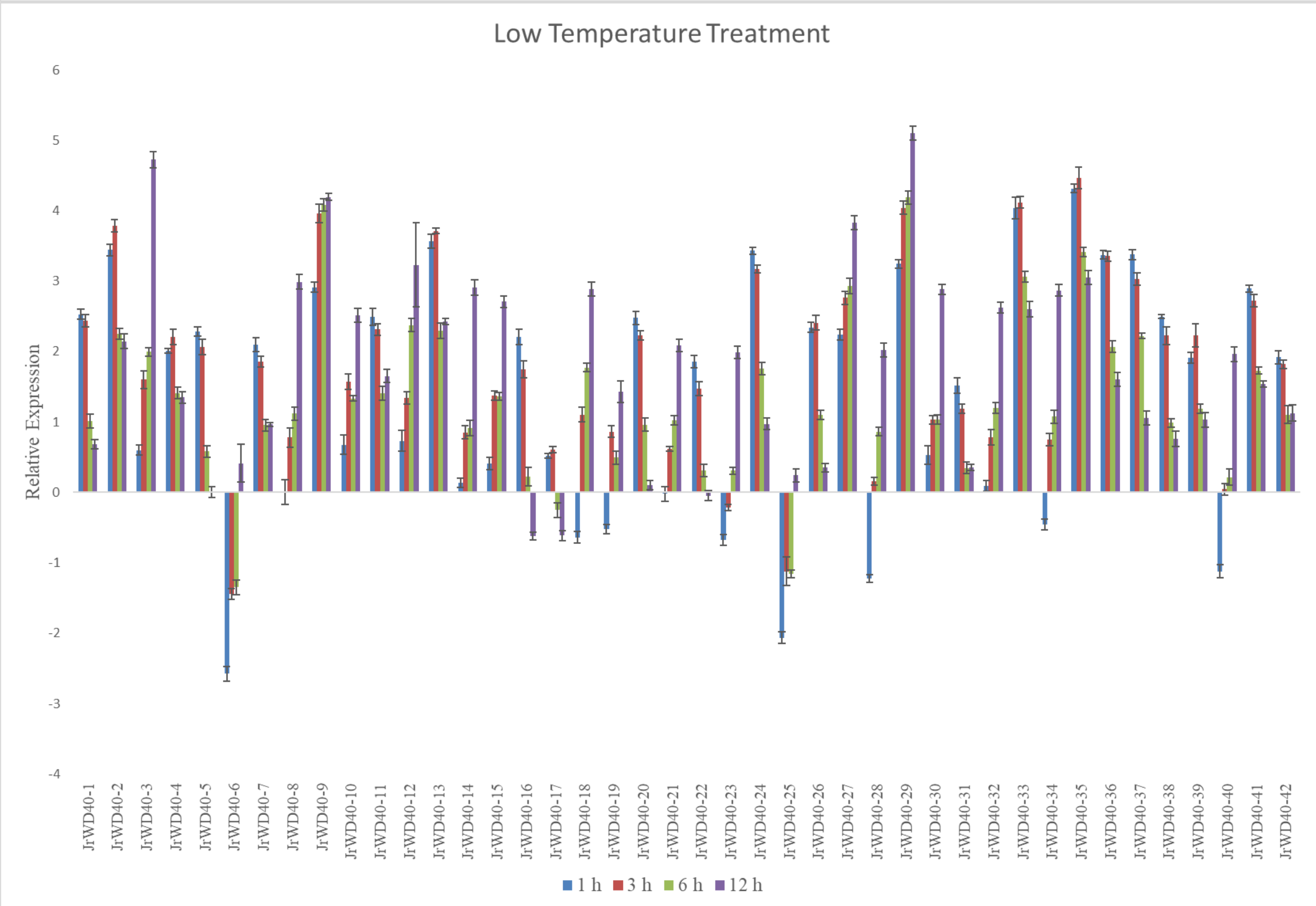
Low Temperature Treatment
Relative Expression
6
5
4
3
2
1
0
-1
-2
-3
-4
JrWD40-1
JrWD40-2
JrWD40-3
JrWD40-4
JrWD40-5
JrWD40-6
JrWD40-7
JrWD40-8
JrWD40-9
JrWD40-10
JrWD40-11
JrWD40-12
JrWD40-13
JrWD40-14
JrWD40-15
JrWD40-16
JrWD40-17
JrWD40-18
JrWD40-19
JrWD40-20
JrWD40-21
JrWD40-22
JrWD40-23
JrWD40-24
JrWD40-25
JrWD40-26
JrWD40-27
JrWD40-28
JrWD40-29
JrWD40-30
JrWD40-31
JrWD40-32
JrWD40-33
JrWD40-34
JrWD40-35
JrWD40-36
JrWD40-37
JrWD40-38
JrWD40-39
JrWD40-40
JrWD40-41
JrWD40-42
1 h
3 h
6 h
12 h
