# Supplementary material for: Identifying and expression analysis of WD40 transcription factors in walnut
Source: Plant Genome. 2022 Jul 29;15(3):e20229. doi: 10.1002/tpg2.20229 (PMC12806941; doi:10.1002/tpg2.20229)
Supplement: Supplementary file 3 [file TPG2-15-e20229-s007.pdf]

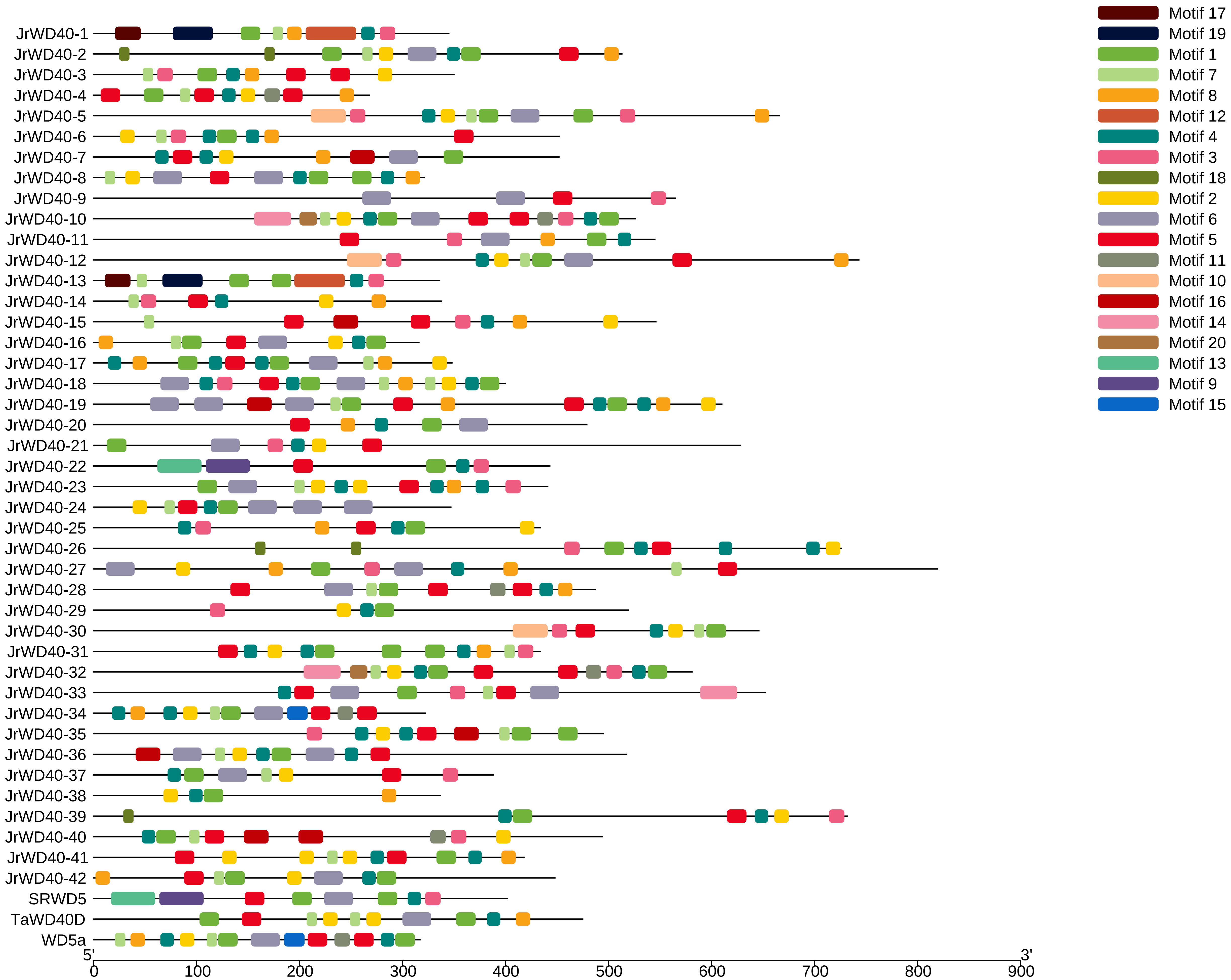

JrWD40-1
JrWD40-2
JrWD40-3
JrWD40-4
JrWD40-5
JrWD40-6
JrWD40-7
JrWD40-8
JrWD40-9
JrWD40-10
JrWD40-11
JrWD40-12
JrWD40-13
JrWD40-14
JrWD40-15
JrWD40-16
JrWD40-17
JrWD40-18
JrWD40-19
JrWD40-20
JrWD40-21
JrWD40-22
JrWD40-23
JrWD40-24
JrWD40-25
JrWD40-26
JrWD40-27
JrWD40-28
JrWD40-29
JrWD40-30
JrWD40-31
JrWD40-32
JrWD40-33
JrWD40-34
JrWD40-35
JrWD40-36
JrWD40-37
JrWD40-38
JrWD40-39
JrWD40-40
JrWD40-41
JrWD40-42
SRWD5
TaWD40D
WD5a
5'
3'
0
100
200
300
400
500
600
700
800
900
Motif 17
Motif 19
Motif 1
Motif 7
Motif 8
Motif 12
Motif 4
Motif 3
Motif 18
Motif 2
Motif 6
Motif 5
Motif 11
Motif 10
Motif 16
Motif 14
Motif 20
Motif 13
Motif 9
Motif 15
