# Supplementary material for: Identifying and expression analysis of WD40 transcription factors in walnut
Source: Plant Genome. 2022 Jul 29;15(3):e20229. doi: 10.1002/tpg2.20229 (PMC12806941; doi:10.1002/tpg2.20229)
Supplement: Supplementary file 4 [file TPG2-15-e20229-s006.docx]

**Table S2 the targets of qRT-PCR products.**

| Gene name | the segment of qRT-PCR products | Target length |
| --- | --- | --- |
| JrWD40-1 | ATCAAGACGGTTCTGACGAGCAGCAGAAGCGGTCGGAGATATACACGTACGAGGCACCGTGGCACATCTATGCCATGAACTGGAGTGTCCGCCGCGACAAGAAGTACCGGCTGGCTATAGCCAGCCTCCTGGAACAGTACCCAAACCGCGTGGAGATCGTTCAGCTGGACGACTCCAACGGTGAGATCCGCTCCGATCCAAATCTCTCCTTTGAGCATCCCTACCCTCCCACCAAGACCATCTTTATCCCGGACAAGGAGTGCCAGAAACCCGACCTTCTCGCAACCTCCAGCGATTTCCTCCGTGTGTGGCGGATCTCCGACGACCACCGCGTCGAGCTTAAGAGCCTCCTCA | 354 bp |
| JrWD40-2 | ATGCGATACTGCCTCAAGTGGCTCAGCTTAAGCTGCCCAGGAATAAATTGGAGGATTTGTATGAGCAGATTGTATTAGAAATGATTGAACTTCGAGAGTTGGATACCGCACGAGCTATTTTGAGGCAAACTCAAGTGATGGGTGTCATGAAGCAAGAGCAACCGGAAAGATATTTGAGACTTGAACATCTCCTAGTCAGAACTTACTTTGATCCAAATGAGGCTTACCAAGATTCAACTAAGGAAAAACGCCGTGCACAAATTGCCCAAGCTCTTGCTGCCGAAGTTA | 288 bp |
| JrWD40-3 | ATCAACGGTGACCTTCACCTCTACCGTTACGGTGCTAATGCCTTGCCTCAAAGGCTTTTGGAAGTTCGCGCGCACACCGAGTCTTGCAGGGCTGTTAGATTCATCGACGGCGGGCGTGCAATTGCGACGTGCTCTCCGGACCGGTCAATTCTCGCTACGGATGTGGAGACTGGATCACCGATTGCTCGTCTTGAAGATGCCCATGGTGATGCAATCAATAGATTGATAAACATAAGCGAGACGACCATCGCATCGGGTGATGATGAAG | 268 bp |
| JrWD40-4 | GTCAGGAGACTATGATGGGGTGGTCATGGAGTATGACCTCGAGAGAAGAATGCCCATTTTTGAGCGGGACGAGCATGGCGGCCGCCGAGTCTGGAGCGTGGACTATTCGCATTGTGATCCGGTTATAGGGGCATCGGGGTCGGACGACGGCACCATGCAGATGTGGGATCCACGATGCGAAGGTGGGAGATGCGTGGGTACGGTGCAGCCCAGCGTGGGGCGTAGTCCCGTCTGCTGTGTTGAATTCAATCCCGTTGCTGGGGCATTAATCGCCGTCGGATGTGCGGACCGGAAGGCTTACGGGTACGACATCAGAA | 317 bp |
| JrWD40-5 | CGTCTGAAGAGTCATCTGTAGAAGAAGAGATAGGTTCTATACATTTAGAATATGGTATTTGGATGAATGAACCACGAAGCATTAAGGAAAGGCGGGAGAATTTCTTACGTGGAATTGGTTTAGCTGAATTTTCTTCTCCTGGGATTTGTGCGGAGATTGGAATGAAAGTTTCTGGCTTGTCAGAGGTGATGGGATTGCAGAGGCTCAAGCAATGCAGTGGAGCTGTCTCAAGCTCTTGCA | 240 bp |
| JrWD40-6 | ACGACATCAAGCTCTGGTTAATAAATTCAGGTGCAGCGCAGACGAAAGGCCCTGCAGCTTCCTATCAAAATAGCCTTTCTTACCACAGTTCTGCTGTAAATGTCCTTCGGTTCTCTCCTTCCGGAGAACAACTCGCATCCGGTGCTGATGGAGGTGAACTGGTAATTTGGAAGTTACACCCCACAGACACAGGTCAAACATGGAAGGTTTTCAAGACTTTATCATTTCACAGGAAGGATGTCCTGGACCTGCAGTGGTCTGCCAATGGTGCATTTCTCATTTCTGGGTCTGTTGATAATTCTTGCATCGTGTGGG | 315 bp |
| JrWD40-7 | TTCTGGCTCAATGGATGGAGATATTCGCCTATGGGATATTGCTTCCAGGCGAACAGTTTCTCAATTTCTTCGTCACCAAGGTGCAGTACGAGGTCTGACAGCATCTACAGATGGACGTGTTCTTGTATCATGTGGCACTGATTGCACTGTCAGGCTTTGGAATGTACCCGTTGCCACTCTTAAGGAGTCATATGAATCTTCTGATAACTCTGTCAAGCCACTGGAAGTTTATGTTTGGAAGAATGCATTTTGGGCTGTTGATCACCAATGGGATGGGGTTTACTTTGCCACAGCTGGTGCTCAAGTGGATATATGGAACCACAATAGGTCTC | 332 bp |
| JrWD40-8 | TCTCTAGACGAGACTGTGAAGCTCTGGAGGTCCGACGAACTGGTCCCGGAGCGCACCAACACGGGCCACTGCCTCGGGGTTGCGTCCTTGGCGGCTCACCCCTCTGGCCTCATCGCCGCCTCGGCCTCTCTCGACAGCTTTGTTCGGGTCTTCGACGTAGATACCAACGCTACCATTGCCACTCTCGAAGCTCCGCCATCTGAAGTCTGGCAATTGCGCTTCAATCCCCAGGGTTCCACTCTAGCAGTTGCTGGTGGGGGTAGTGCGTCAATCAAGCTTTGGGACACTGCCACGTGGGACTTGGTTGCCACTCTA | 315 bp |
| JrWD40-9 | AGGAAGAGGATGAGAAGGCAATTGAGCAAGACAGAGAAATGAAGAAGCTAGAGAACTCTTTGTTCGGGTCCCTGTATTCACCTGTTGAGTTTGGGAATGAAGACGAGGAAGACACTCGAGACTGGGCTGAGAATGGTTCTGCTTTGTTCATCGTGGACCGAACTGCAAATGGTGTGCTCTCTGTTTATGAAGAGGATGCAGAATTCCCA | 209 bp |
| JrWD40-10 | GATGGTTGCACTGATACCCTTAATGAAATTAAGGTGATGGATGAGACAAGAGCTTCTGCTTTGTTTATTGTATTCAAACAGTGCTTATTGGAGTGCTTGAATCATGGGGATGATGCCATGGCTTTGGCCATTTTAAGAAAAGTGGTTTCTGCATTAAGGTTGGGCAAAGACAAGGCTCACAGCCTTGCTCAGAGTATCCTCTCTTTGAGGGATACAGAGTTTGGTAAAATGGATGATAATATTGTTCAGGAATTAAGAAAGAAGTCGTTGGTGGAGTT | 278 bp |
| JrWD40-11 | ATCTCAGCTTGAGGAAGCAGAGAGAAGGTTGCATGATTCACAATCTAAATTGGCTCGACTTCGAAGCCAAAGTAATGTTGTGCCAATAAAAGGTTCTCTGGATAATGCATCGAAGAACGTGAAGGTGGAGCGCAGATCAACTAGTCCCATACATATAAATGGAGGTTCTTCTAGGAACCAGCCTCAGTCAAAACCAGAACTTTTAATCCCTGCTGCGAATCCAAAACTTTCCCAGCCTATAAAATCTTCAAATATTTCTAAGACTCAAGCCAATCCAC | 278 bp |
| JrWD40-12 | GGTACTGAGAACTTCAGGTTTTGAAGAAGGGTGTCCATTAAGTAAGTGTACCATGTCAGATGAAGTGTCACATAAGTTAGAAAATGGTGTTTCAGAAGATAGTATTATGTGTACAATCAGGAATTTGGATGATGGAACCGAGTTCCTTGTGGACGAATTGGATCGCAATGGAATGCTTAGCAGACTACGTGAAGTGCGTTCCAATCGATCAGTGAGTTTTGAAGAATTTCAGAGAATTTTCGGGCCATCTCCTTTGGTTCAGCAGCACTTGCAGAGAGAAG | 281 bp |
| JrWD40-13 | CCATGACAAGAACACTCGCCTCGCCATAGGAAGCTACCTCGAAGACTACAGCAACAAGGTGGAACTGGTTCAGTTCAACCATGCCACCTCTGATTTCACCACCGACAGCCGCCTGGTCTTCGACCACCCATATGCAGCCACAAACCTCATGTTCTTCCCCTCCGAGGACACCATGAATCCCGACATCCTAGCCACGTCCGGGGACTATCTGCGGCTGTGGCAAATCCACGACGACCGCATCGAGCTCAAGGCCCTCCTCAACAGCAACAAGAGCAGTGA | 279 bp |
| JrWD40-14 | ATGAGTCTGATAGCAGGCTCGTACGAGAAATACATATGGGGCTTCAAGCTCAAACCACTTGAAACCCCGTACCTCGCCCTCACCCCTCTTTTCTCGTACCCATCCCACCAATCCCCCATCACTACCGTCGCCGCATGCGGCCACGTCGCTGCCTCTGGCGGCGCCGACGACACCATCCACCTCTACGACCTCCCCTCTGCCTCCTACCTTGGCTCCCTCCACGACCACTCCTCCTCCCTCACCTCCCTCTCCTTCTACTCCCCTCCCAACCTTTCCTTCCCCCGTAACCTCATCTCTGCCGCCTTCGACGGCTCCGTTTCTATCTTCGACGCCGACCCTTTCGTCCTCCTCAAGTCCATCCGCCCCCACAGGAAGGCCATCAACGA | 386 bp |
| JrWD40-15 | TACAAGCTCCACTACGAGAAGACCCACCCCTCCAGTCTCCTTCATTACGCCCATGGAAAAACCGTCACTCAGGTAACTCTAGCACATCTAAAGGACAAGCAGGCCCCATCAACCCCACCAGCCCCTCCTTCAAGCTTCAGTGCTAGCAGTGGAGTGCGATCAGCTGCAGCAAGGTTATTGGGTGGAAGTAATGGGAGCCGTGCCCTTAGCTTTGTTGGAGGGAATGGTGGCAGTAAGA | 238 bp |
| JrWD40-16 | GATGTCAACTCTAGCAGCCCTCAACCAGTGATGAGCTATGATTCACATACTAATAATGTGATGGCAGTGGGGTTTCAATGCGATGGAAATTGGATGTATTCAGGTTCTGAGGACGGCACAGTAAAGATTTGGGATTTGAGGGCTCCAGGTTGTCAAAGGGAATATGAAAGCCGAGCAGCTGTCAACACTGTTGTGCTCCACCCAAACCAGACCGAACTTATATCAGGAGACCAAAACGGGAATATTCGTGTGTGGGATTTAACAGCTAATTCATGCAGCTGCGAATTGGTGCCAGAGGTCGATACTGCTGT | 311 bp |
| JrWD40-17 | TGGAGTTGTACGAAGGAGGGTGCGAGCTGAAGCTGCTCGAAACGCTGGAAGGCCACAGCGACAGGGTTTGGAGCCTAGCCTGGAACCCAGTCACCGCCGCCGCGGGGACCCCTCTCGTCTTTGCCTCCTGTAGCGGCGACAAGACCGTCCGAATCTGGGAGAACACTCCCTCCCACTCGGCTTCTTGGACCTGCAAGGCAGTTCTGGAAGACACGCACACCAGGACCGTCCGATCTTGCGCTTGGTCGCCGTCGGGGAAGTTATTGGCAACCGCGAGCTTCGATGCCACCACTGCCATTTGGGAAAACGTTGGTGGTGACTACGAA | 326 bp |
| JrWD40-18 | TGAGGAGCCTGATGATTCCGTGCATATATTCACCGGTCATACCGGTGAGCTTTATACAGTTGCCTGCAGCCCAATGGATGCTTCGTTAGTGGCAACTGGGGGTGGAGATGACAAAGGATTTCTTTGGAGGATTGGGCACGGAGATTGGGCTTTTGAGCTCCAAGGTCATGGAGATTCCATTTGCTGTTTAGCCTTTGGCACCGATGGACAGTTGCTGGCATCTGGATGCTTGGGTGGACTTA | 242 bp |
| JrWD40-19 | ATGACTCAACTCGCAGAGACCTACGCGTGCGTCCCAACCACCGAGCGCGGCCGGGGGATCTTGATCTCCGGCAGCCCTAAATCTAACACCATCTTATATACTAATGGCAGATCCGTGATCATCCTCAACCTCGACAACCCTCTCGACGTCTCCGTCTATGCCGAGCACGCCTACCCTGCCACCGTCGCCCGATTTTCCCCCAACTGCGAGTGGATCGCCTCCGCCGACGTCTCCGGGGCCGTTCGAATCTGGGGGACGCGCAACGAGTTCGTTTTGAAGAAGGAGTTCAAGGTCCTCTCCGGTCGGATTGACGATCTCCAGTGG | 324 bp |
| JrWD40-20 | ACCGAGACTCCAATCGTTATCCGACGGTCCTTGCGTACGCGTGGGATGCCGCCTGATTACTCTGACTCCAAAGGTCTTGGCGATATTGTTGAAGCAGCCGATAAGGTTTCCAAATCTCCGACCTCGTCGAAGCCTTCGCCGCGCGCCTTGGGCCCTCTTAGCGTGAGAGATGCGTATCGTGGAACTGGGTCTGATA | 196 bp |
| JrWD40-21 | CAGACATCACCTCAACTGATGAGATAAATATATTAACCAGACATGAAAGAGGGCACCTATCAGTTGATTATACATGCATGAAGTGGTTATCGTTGGACAGAAGGAGAAAAAGGAAGCTTGGTTACTCATTGTTAGTCTTAGGAACAGGTAGTGGTGATATTTTAGCACTGGATGTCTCTGCTGGTCAATTGAAATGGAGAATTAATGACTGCCACCCTGGGGGTGTTAATTCCATTTCATCTCCTACGAATGGTTCCTGTATTTATACTGCAGGTGCTGATGGCATGTT | 289 bp |
| JrWD40-22 | TACATGGTGGATGAGCAGGAAATGGAAGATTTCGGTCATGAATTCGAGGAAGAAGTCTATGGTGGAGATGGTGGAGAGTTGGGTCTTGACGAGTATAACATGCTGACCAGAGTGACAGATACCTCGGCTGCCCAAGCCAGGAAGGGAAAGGATATTCAAGGTATACCATGGGATAGACTTAATATAACGAGGGAGAGCTATAGGTTGACGAGGCTCGAACAGTACAGGAA | 230 bp |
| JrWD40-23 | CAGATCCTGTCCATTGCTGTGTCCAACGGCTTCGTCTACACAGGCTCGGATGCCAACGCCATCAGGATTTGGAGGCTCCCGGAGTTCGCCGAGTGTGGGCAGCTCAAGACCAAGGCTTGCATGGTGGTTGCATTGGTGGTGTACAATGAAAGAGTCTATGCAGCCTATGGTGACGGCAAGATTCGGGTGTGGCGCAGAACATCTGATCATGGGGGCTTGAAGCACATCCGATTGGCGACGATCCCCAAGACTGGAAGTTATGTCCGGAGCTATATTGCCGGCAAAGATAAAACGATGAAGCATACAGGACCCATAACGGCACTTGCGATCAATGCA | 336 bp |
| JrWD40-24 | ACCTGGTTGCTACTTCGTGGGATAACCAGGTAAGATGTTGGGAGATAATGCATAGTGGGGCTGTTGTAGGCAGTACACCTAAGGCTTCAATATCTCATGACCAACCGGTGTTGTGCTCGACTTGGAAAGATGACGGAACAACTGTATTTTCTGGAGGTTGTGATAAGCAAGCTAAAATGTGGCCTTTGTCTGGTGGGCAGCCAATGACTGTGGCCATGCATGATGCACCTA | 231 bp |
| JrWD40-25 | CCATCCAACAGCAGTTGCATTCTCTGATGATGTGTCTTCTATAGTCATAGCTTCTCAGACGCTTACTGGTTCTTCTTTGTACATGTACGGAGAGGAAAAGCCCAAAGGTTCTAATGAGAATAAACAAGCCAAGCTTCCTCTCCCAGAAATTAAATGGGAACATCACAAAGTCCATGAGAAAAAAGCTATTATAACCCTTGTTGGCACTACTGCAAGTTATGGAAGTGCTGATGGAAGTAC | 240 bp |
| JrWD40-26 | TTCTCTGGATGATGCAGCAATAAAGCAAAGATTTGGGGACAATGTGAGCCAGCTTTTGGATCCAAATCATGCCTCGATATTGAAGTCAGCTGCAGCAACTGGCCAGCCTTCAGGGCAACTGTTGCATGGCACAGCTGGTGGGATGTCTCCACAAGTTCAAGCTCGCAATCAGCAACTACCAGGGTCCATGCCGGATATCAAGAGTGAGATTAATCCGGTTTTAAATCCCAGAGCTGCAGGTCCTGAAGGATCAT | 254 bp |
| JrWD40-27 | AGACGGTTCGCTTGAGATTTGGCTCGTCTCTCCCGGCTCCGTCGGCTGGCACTGTCAGCTGACCATCCATGGGGACCCTAATTCCAGGGTTTCGTCGCTACTGTGGTGTCGAGCTGGTTCAAAAGGCTTGCCATGTGGTCGGCTATTTTCGTCAAGTATTGATGGTTCGGTTTCGGAGTGGGATCTTTTTGATTTGAAGCAGAAGACTGTTTTAAACTCAATTGGAGTCTCAATCTGGCAA | 241 bp |
| JrWD40-28 | AGTGAAGATGTTGGCTGGTCGAGAGTGTAATTATTCAAGAAGGGGGAGATTCTCATCAGCCGATTGTTGTCATGTCCTAAGCAGATATCTGCCAGTAAATGGTCCTTGGCTAGTTGATCAACAGACCAGCCGAGCCTATGTCTCACAGTTTTCGGCTGATGGTTCCCTTTTTGTTGCTGGATTTCAGGGAAGCTACATTAGGATATACAATGTGGACCGAGGATGGATGGTTCAGA | 236 bp |
| JrWD40-29 | AGGAGATGTCTGAGGAGTTAGATTTGAAAATGGAGAAGTACCTAAGAGGCGGTGCTGCTAACTTGGAGGTTCTACAAGATAAAAAACTCAAAGGTCAACTTGCTCATAGAGAAAACTTGTATGGAAAATCTGCTAAGGCTGCTGCTAAGTTTGAGAAGTGGCTTATGCCAAGCGAGGGGGGTTATCTGGAGGCTGAAGGTCCAGTAGAGAAGACATGG | 218 bp |
| JrWD40-30 | AACGAGTGCTTCTACGAGTCCCTTGACCGCATCGTCTCGTCATCCTGCTCATGCTCCAACTCCAACTCCGACACCGAAGAGAACGACGCCGTTTCTTCTTCGGCCTCGCCTAATCATGGGTCGGTACCGAAATTCCCAATGGGCGCGTCCAACAAGTACGATGTCTGGATCTCCGAGCCCTCCTCCGTCTCCGAACGCCGCTCGTGTCTCCTCCGCCAAATGGGCCTCGGCAACGACCCCTCCTTGTCCCCCGCCAAACCCACCCGAGATTTCGGATTCGGTGGAGACATTGGCCGATCAGTGTCGTCCGACCGCTTGTCCAATCACTCCGGTGCTTCCACCATGGTTCGAT | 352 bp |
| JrWD40-31 | CATCGATTGCAATCCCTCCGAACCTCACCAGATTGGGCCTCTCCACCCTCGTCAACAATTTACTCCAATCCGGAAATCCTGATTGGAAACTTGAGCCTTTTGATTTTCTAATCGATGGCGAGCTGGTGCGGATGTCACTTGAAAAGTTCCTTCTTGCCAAGGGCATTTCGGCGGAGAAGGTATTGGAAATTGAATACATAAAGGCTGTGGCCCCACGGAAAGAAGAAGAACCTTCTTTTCATGATGATTGGGTCAGTGCAGTCGATGGTTCT | 272 bp |
| JrWD40-32 | ACAGTGGTGCTCATCTAGAGGAAGAGTCTGGGATACCCTTACACTCCTCTGTGGTAAATCTGTTTATGCAGCAAATACTTGATGGCAATTGGGATGAAAGTGTTTCTACATTGTGCAACATTGGACTGTTGGATGAAAGCGTTGTTAAATCAGCTTCTTTTCTGATTCTGGAGCAGAAGTTTTTTGAACTTTTGGATGCAGAAAAGGTCATGGATGCGCTAAATACATTGAGGACTGAGATTGCACCT | 248 bp |
| JrWD40-33 | ATGAGGATGAGGATGAGGATGGAGGCGTGATGATTGGTCCGCCGAGGCCGCCGCCGGCCCAATCAGGTTCCGACGATGAGGAGAAGGAGGAGATGATTGGACCGCCGAGACCGCCTCCTGGACTGAATGTGGGCGATTTAGGTTCGGACGAAGAAGAGGAGGAGAATCGGTACCGGATTCCGCTAAGTAATGAGATTGTTCTCAAGGGACACACCAAGGTTGTTTCAGCTCTTGCAGTTGACCACTCT | 248 bp |
| JrWD40-34 | TCCAACTCCGACTCCAATGCCGATCCCACCGTCTCTCCCCTCCAAGAATTCCTCGGCCACGAGCAGGGCGTCTCCGACCTCGCCTTCTCATCTGATTCGCGTTTCCTCGTCTCGGCCTCCGACGACAAGACTCTCCGGCTCTGGGATGTCCAGACGGGGTCCCTCGTCAAGACCCTCGCTGGCCACACCAACTACGTCTTTTGCGTCAACTTTAATCCACAGTCCAACATGATCGTCTC | 239bp |
| JrWD40-35 | AAGTGGAGATGGTGACAGCGAGGAAGATGATGAAGATATGAATGTTGATGCTGCCAAGGAAGCTGATGAAGCTGCCCATGCATTGGAGGTGGCGGATGCACTTGGAAGAACTTCCAATAACACAAATTCAGGGACCGGTTTTGATGATGTAACCAACAGTTTGAGGGAACTTGACATGGAACATTATGATGAGGAGGATGATGGCATTGAGTTATTTAGCACAGGTCTAGGGGACGTCTACTACCCAAGTAATGACATGGATCCATATATAAAAGATAAGGATGATGAGGACTCTGA | 297 bp |
| JrWD40-36 | AGAGTCCAAGTACTGGTCCTCCTTCAAAACCCACCAAATCCCAAACCTCATCTCCTCTGTCACCTCCCTCACCTTCTCTCCCACGACTCCACACCCCTTCGCCGCCACCTACTCGACCTCGCTTACCCTCTTCAGCCCACAGACGCTCTCTCCCTCCTCCACTATCTCCTCTTTCTCCGACGTCGTTTCCTCCGCCTCCTTTCGCTGTGACGGCCTCTTGATTGCCGCCTCGGATCTCTCCGGACTAATTAAAATATTCGATGTCAAAACCCGGACCCCACTGCGTAAACTCCGGTCCCACACACGCCCGGTGCGTTTTGTCAAATACCCAGTTCATGATAAGCTCCATTTGGTCTCTGGCGGCGACGATGCCCTCGTTAAGTACTGGGATGTCGCCACCGAGACACCAATATTAGAGCTTCAGGGACATAAG | 433 bp |
| JrWD40-37 | GAAGCTGTACTCACCAGTAACTGGTCAGTACTACGGAGAGTGTAGGGGTCACTCTACGACGATAAATCACATCTCATTCTCGGGTGCATCAACCCCACATGTATTGCACTCTTGCTCTTCTGATGGAACCATTAGAGCTTGGGATTCGAGGTCATTCCAGGAGGTTTCATGTATAAGTGCTGGCAATTCGCAAGAGGTCTTCAGCTTCTCGTTTGGAGGATCAAATGATAACCTTCTTGCTGCAGGATGTCAA | 253 bp |
| JrWD40-38 | AAGGAGTCACTAAGACGCCTTTCAAGCATAACCTGGATGGCAGAGGAATTGCACAAAGTAACCAACTATTACCAACCGAACAAATCACGTATTTCTTGTGTTCACACACAAATCATGGAAGCGGGTCTCAAATGCAATGGAGCTGTATGTGCCCTCATCTACTACAAGGGACTGCTTTACAGTGGATACTCAGATGGTTCAATCAAGGCGTGGGACATCAAGGGCCAATCAGCCATGCTTGTATGGAACATGAAGGAGCACAAGAAGGCAGTGACATGCTT | 281 bp |
| JrWD40-39 | AGAGGACGAGGAATGCTTCAAATCCTCAATCCGAAGAAGAGCCTTTAATCAATGAAAAACCTTCACAAGCACTACAACAAAAACAGCATCAGCAGCAATTAGCATCTGCTTCTGACTCTGAATCTGATTCTGACTACGAGGATTCTTCAGAGGCTGGTGACTCTGGTCAATCGTTGAGTGAATCAGAAGCCAGTGAAAGTGGATCGTCTGGAGATGAGGGCATTGCCAATTCAGATGATAATGGCTTGGACGACGAT | 257 bp |
| JrWD40-40 | ATGCAGATGGTGACGTTCTGGTTTCAGGATCTGATGACAGGCGGGTTATACTCTGGGATTGGGAGACTGGGCGTGTCAAGCTTTCGTTTCATTCAGGTCATAATAACAACGTTTTTCAAGCAAAGATCATGCCTTACACAGATGACCGAAGCATTGTTACCTGTGCTGCAGACGGGCAGGTGAGACTTGCTGAGATTTCGGAATGTGGAAGAGTGGAAATGAAATTGCTTGCTAAACATCAAGCACGAGCTCAT | 254 bp |
| JrWD40-41 | GTTCGTTGACGGATACAGCGGTAGCTATAAACAACGGGGCGGTTAAAGCTCTGGTAGTCTTGGAGGATAAACTATTCACTGCCCATCAAGATCACAAAGTCCGTGTGTGGAAGACCGATAACAATACCCAAAACCAAAAATACACATGCATAGCCACTCTCCCCACGCTTAATGATCGTTTTTCAAGGCTTTTCTCGGCCAAGAACTACGTGGAGGTGCGGCGGCACAAGAAGTCCACGTGGGTACATCACGTT | 254 bp |
| JrWD40-42 | AAGTGGTAGGCAGCTTAGAAAATGGCATGCACATTATAGGGCTGTTACTTGCTTGGTATTTTCGGATGATGACTCGCTGCTAATTTCTGGTTCAGAAGATGGATCCGTTAGAGTTTGGTCCCTTTTCATGATGTTTGATGATATTAGACAGCAGGAAGCTAGTCATCTATATGAGCACAATTTTTCAGAGCACACTTTGCGTGTAACTGATATTGTCACAGGATATGGAGGA | 232 bp |
